# Bacterially-Associated Transcriptional Remodelling in a Distinct Genomic Subtype of Colorectal Cancer Provides a Plausible Molecular Basis for Disease Development

**DOI:** 10.1371/journal.pone.0166282

**Published:** 2016-11-15

**Authors:** Katie S. Lennard, Ryan W. Goosen, Jonathan M. Blackburn

**Affiliations:** Institute of Infectious Disease and Molecular Medicine & Department of Integrative Biomedical Sciences, University of Cape Town, Cape Town, South Africa; Baylor University Medical Center, UNITED STATES

## Abstract

The relevance of specific microbial colonisation to colorectal cancer (CRC) disease pathogenesis is increasingly recognised, but our understanding of possible underlying molecular mechanisms that may link colonisation to disease *in vivo* remains limited. Here, we investigate the relationships between the most commonly studied CRC-associated bacteria (Enterotoxigenic *Bacteroides fragilis*, pks+ *Escherichia coli*, Fusobacterium spp., afaC+ *E*. *coli*, *Enterococcus faecalis* & Enteropathogenic *E*. *coli*) and altered transcriptomic and methylation profiles of CRC patients, in order to gain insight into the potential contribution of these bacteria in the aetiopathogenesis of CRC. We show that colonisation by *E*. *faecalis* and high levels of Fusobacterium is associated with a specific transcriptomic subtype of CRC that is characterised by CpG island methylation, microsatellite instability and a significant increase in inflammatory and DNA damage pathways. Analysis of the significant, bacterially-associated changes in host gene expression, both at the level of individual genes as well as pathways, revealed a transcriptional remodeling that provides a plausible mechanistic link between specific bacterial colonisation and colorectal cancer disease development and progression in this subtype; these included upregulation of *REG3A*, *REG1A* and *REG1P* in the case of high-level colonization by Fusobacterium, and *CXCL10* and *BMI1* in the case of colonisation by *E*. *faecalis*. The enrichment of both *E*. *faecalis* and Fusobacterium in this CRC subtype suggests that polymicrobial colonisation of the colonic epithelium may well be an important aspect of colonic tumourigenesis.

## Introduction

The association between specific bacterial species and colorectal cancer (CRC) has been widely reported and, based on mechanistic *in vitro* data, is generally believed to play at least some role in cancer initiation and/or progression. However, the molecular changes in host cells that may link colonisation to disease *in vivo* remain relatively poorly understood. Bacterial 16S rRNA profiling of paired tumour and normal CRC biopsies revealed that while only 3% of biopsy specimens from healthy controls contained any type of bacteria, ~90% of patients with adenomas or carcinomas had bacterial counts of 10^3^–10^5^ CFU/μl in both malignant and macroscopically normal samples [1]. This clearly demonstrates increased susceptibility to colonisation of the normally sterile colonic epithelium in these patients—not only in existing tumour tissue, but also in the surrounding macroscopically normal tissue. Whether or not this is indicative of a pre-existing risk to colonisation/infection (i.e. before CRC development) in these patients or instead disruption of mucosal barrier function in macroscopically normal tissue surrounding the tumour, remains unknown.

Plausible bacterially-driven oncogenic mechanisms in CRC include activation of Wnt signaling (ETBF, Enteropathogenic *Escherichia coli* (EPEC), and Fusobacterium), pro-inflammatory signaling (*E*. *faecalis*, *S*. *gallolyticus*) and genotoxicity (EPEC and adherent-invasive *E*. *coli* (AIEC)). The potentially oncogenic features of these bacteria, as well as suspected bacterial components implicated in CRC, have been described previously [2]. However, despite the growing body of research on CRC-associated bacteria and their relationship to various clinicopathological features of CRC, we currently have little understanding of how these otherwise well-studied bacteria relate to CRC transcriptomic patterns, pathways and genomic subtypes *in vivo*. Given the increasing knowledge regarding pathogenesis and clinical outcome that has been associated with particular subtypes of CRC, an important next step therefore is to understand bacterial colonisation patterns within this framework, since this should enable important gene and pathway-level associations to be identified that could underpin new hypotheses on the possible causal roles of these bacteria in CRC.

The existence of genomic sub-types of cancers, including CRC, is now well established in the literature [3–6]. However, the relationship between genomic subtypes of CRC, their pathway features and CRC-associated bacteria has not previously been reported. In earlier work, we quantified the most commonly studied CRC-associated bacteria (Fusobacterium, *Streptococcus gallolyticus*, *Enterococcus faecalis*, Enterotoxigenic *Bacteroides fragilis* (ETBF), EPEC, and afaC- or pks-positive *E*. *coli*) in paired tumour and normal tissue from 55 CRC patients. With the exception of *S*. *gallolyticus*, we detected all these bacteria in both tumour and normal samples at varying frequencies [2]. Here, using these same samples, we investigate patterns of specific bacterial colonisation in relation to genomic subtypes of CRC, including the distinctive gene expression and pathway features that characterise these subtypes, with the goal of identifying potential mechanisms of bacterially-driven tumourigenesis. Importantly, the individual tissue samples utilised for genomic analyses in the present study form a sub-set of the fresh-frozen, paired tumour and normal tissue samples utilised in our earlier bacterial profiling work [2]. The methodology utilised for the bacterial identification and quantification in these tissue samples has been described in detail previously [2] and is therefore not described again here.

**Fig 1:**
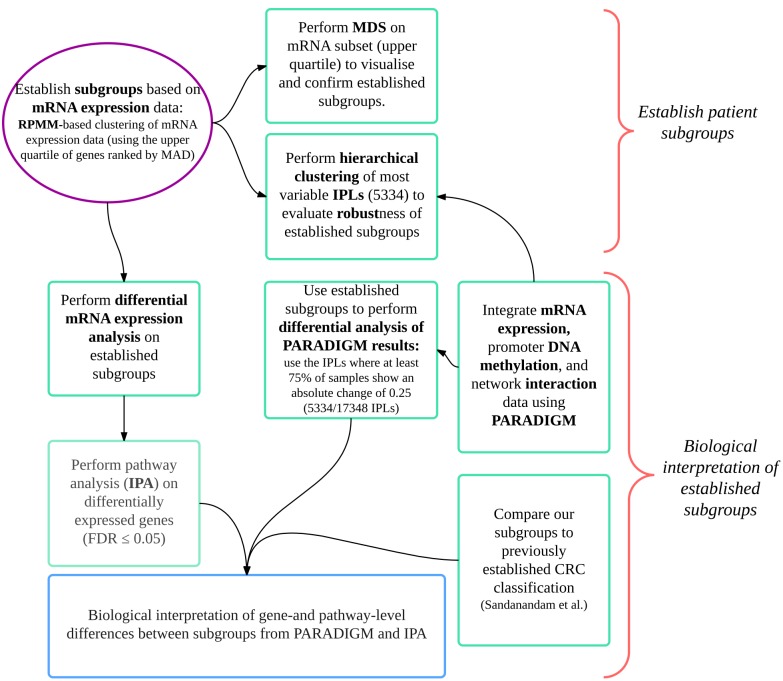
Workflow of CRC subgroup classification and biological interpretation thereof. This workflow was applied to our cohort as well as to an external cohort of 155 CRC samples (for which mRNA expression profiles (GSE13294) and MSI status were available). Numbers indicated on the figure relate to our cohort (N = 19). Median absolute deviation (MAD); integrated pathway level (IPL); colorectal cancer (CRC); Ingenuity Pathway Analysis (IPA).

Unsupervised clustering of Affymetrix Gene ST 1.0 based transcripomic data was used to define CRC subtypes in 19 adenocarcinomas. PARADIGM [7] and Ingenuity Pathway Analysis^™^ analyses of whole-genome gene expression and methylation data, together with qPCR-based bacterial quantitation data, were used to investigate plausible etiological bases of these subgroups and bacterially-associated changes in host gene expression were identified. Our *in silico* workflow was also applied to a well-defined publically available CRC gene expression dataset (GSE13294) comprising 155 colorectal adenocarcinomas to evaluate the relevance of our results in a larger cohort. A summary of this workflow is presented in Fig 1.

## Materials and Methods

### Sample collection and storage

Paired colorectal patient samples (diseased tumour tissue and adjacent healthy gut epithelial tissue) were collected during surgical resection of previously untreated patients at the Groote Schuur Hospital, Cape Town, South Africa. Samples were collected under supervision of the surgeon performing the resection and tumours were confirmed as adenocarcinomas by an independent pathologist. Collected samples were frozen immediately in liquid nitrogen and stored at -80°C. Frozen samples were transitioned to RNAlater-ICE (Ambion), an RNA stabilisation solution, using dry ice to prevent thawing of the tissue at any stage. RNA was extracted using a Dounce homogenizer and the AllPrep DNA/RNA/Protein kit (Qiagen) including DNAse treatment. Ethical approval was granted by the University of Cape Town Human Research Ethics Committee; approval number UCT HREC 416/2005. All participants provided written informed consent to participate in this study; the University of Cape Town Human Research Ethics Committee approved both this consent procedure as well as the specific consent forms used. Participant-level characteristics are listed in S1 Appendix.

### MSI testing and bacterial quantification

MSI testing was performed using the Bethesda panel of microsatellite markers. All primers used for bacterial detection, and their limits of detection (LODs) and qPCR efficiencies, as well as the bacterial strains used as positive controls were previously described [2].

### Microarray-based transcriptomic analysis

Transcriptomic analysis was performed for 19 tumour samples, on Affymetrix Gene 1.0 ST arrays, as previously described [8]. Importantly, the individual tissue samples utilised for genomic analyses in the present study form a sub-set of the micro-dissected, fresh-frozen, paired tumour and normal tissue samples utilised in our earlier bacterial profiling work [2]. Due to variable RNA integrity we devised a method to effectively assess array-quality, and account for known or unknown sources of variation, such as array quality- and batch-effects to allow inclusion of these arrays in downstream analyses [8]. Array data was submitted to ArrayExpress, with accession number E-MEXP-3715.

### Microarray-based methylation analysis

Whole genome array-based methylation analysis was performed on Illumina HumanMethylation 450k BeadChip arrays, according to the manufacturer’s instructions (Illumina 2011), as described in detail in the S2 Appendix. Array data was submitted to ArrayExpress, with accession number E-MTAB-3027.

CIMP status was defined using the Hinoue et al. [5] CIMP-defining marker panel *(B3GAT2*, *FOXL2*, *KCNK13*, *RAB31*, and *SLIT1*) that identifies CIMP+ (CIMP-H or CIMP-L) tumours with 100% sensitivity and 95.5% specificity, with 2.4% misclassification using the condition of DNA methylation of three or more markers with a ß-value threshold of ≥ 0.1 (see S2 Appendix for more detail).

### CRC subtype classification

Tumour subtypes were classified using recursively-partitioned mixture model (RPMM) clustering [9]. RPMM was applied to the third quartile most variable gene expression data by median absolute deviation (MAD); a Gaussian distribution was specified to suit the distribution of gene expression data. For our cohort, the gene expression data used for RPMM was adjusted for batch and quality factors using the ComBat algorithm [10], as previously described [8]; disease status (tumour vs. normal) was specified as the phenotype of interest. Multidimensional scaling was applied to the subset of transcript clusters that were used as input for RPMM, to visually explore the underlying relationship between samples, and to validate the RPMM-based subgroups. Identified subgroups were further assessed at pathway-level using PARADIGM [7] as described in S2 Appendix.

### Statistical analysis of bacterial subgroup associations

Bacterial subgroup associations (in terms of presence/absence of each bacteria) were evaluated using Fisher’s exact test; in the case of Fusobacterium, where the vast majority of samples were positive, subgroup associations were evaluated in terms of ‘no-colonisation’ or ‘low-colonisation’ samples vs. ‘high-colonisation’ samples, where quantitative data (copies/50ng) were log2 transformed and samples with no-colonisation were arbitrarily set to 1 before log2 transformation; the third quartile (calculated across colonisation-positive cases only) was used to discriminate low- and high-colonisation cases, as previously described [2]. Similarly, the frequency of high-colonisation by any bacterium (colonisation-H) was also compared between subtypes.

### Differential gene expression and pathway analyses

Differential gene expression analyses were conducted using the R package limma. For differential gene expression analyses by RPMM-derived subgroup (group B vs. A) analyses were conducted on gene expression data that had been corrected for batch and quality factors using ComBat, while specifying the RPMM-subgroups as the phenotype of interest (as opposed to disease status). This allows conservation of biologically meaningful subgroup-specific variation, while adjusting the data for known sources of technical variation.

To identify gene expression changes specific to each bacterial species quantified, ComBat-based batch and quality correction was performed for each bacterial comparison individually, by specifying the comparison of interest in the model. Differential expression analyses were performed on transcriptclusters that mapped to Entrez Gene Symbols, thus excluding control probes and transcripts with poor annotation, leaving 21934 of the original 33297 transcriptclusters. Differential analyses by each bacterial species were conducted separately in tumour and normal samples and the comparisons made were: a) samples with vs. without colonisation by a particular bacterium and b) samples with high vs. low/no-colonisation by a particular bacterium. Comparisons were only made where at least three samples per group were available, as summarised for tumour and normal samples in S1 Appendix.

Ingenuity pathway analysis (IPA) was applied to the subset of significantly differentially expressed genes (FDR ≤ 0.05 and an absolute fold change ≥ 1.25). For each cohort genes significantly altered between subtypes were used to investigate the IPA categories: canonical pathways, upstream regulators and diseases and functions.

PARADIGM-derived IPLs were also compared between subgroups (using limma) (FDR ≤ 0.05 and an absolute difference in median activity score between groups of at least 0.25 were deemed significant).

The CRCassigner-786––the gene-signature subtype-classifier proposed by Sadanandam et al.––that defines their five CRC subtypes [3], was applied to our transcriptomic data to evaluate our subgroups in the context of these gene signatures. To apply the CRCassigner-786 to our cohort, each of the 786 genes in the panel were assigned to the Sadanandam subtype that had the maximum Prediction Analysis of Microarrays (PAM) score (published by [3]) for that gene. Hierarchical clustering (Euclidian distance, complete linkage) was applied to the gene expression data for each subset, to evaluate which of our samples most closely resembled a given subtype.

## Results

### Specific bacterial enrichment in distinct transcriptomic CRC subtypes

Numerous of different bioinformatics methods have been utilized in the literature over the years to cluster biological samples based on transcriptomic datasets. Here, we chose to use the well established recursively-partitioned mixture model (RPMM) approach [10] in an unsupervised mode to cluster our CRC samples into genomic subtypes based on our transcriptomic data. RPMM is a mixture model approach commonly used for cluster analysis of methylation data, which can also be applied to gene expression data by specifying a Gaussian distribution. No gold-standard currently exists for clustering of gene expression data. We selected RPMM because it allows for more direct comparison between transcriptome- and methylome-based subtypes (data not shown) and because mixture model clustering has been shown to outperform standard methods of hierarchical clustering [11]. Further, previous studies have shown good concordance between CRC subtypes established using different clustering techniques [12].

**Fig 2:**
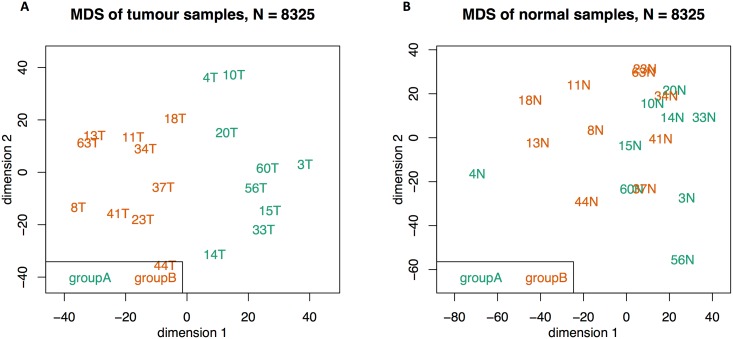
Multidimensional scaling of the top quartile (N = 8325) most variable transcript clusters in tumour (left figure) and normal (right figure) samples. The RPMM-derived groups A and B are highlighted in green and orange, respectively. The tumour-derived subgroups are also highlighted in the adjacent normal samples (right figure) to evaluate the level of agreement between clustering of normal samples with the tumour-derived subgroups.

We identified two main groups by RPMM clustering, one of which had two subgroups that were combined for downstream analyses We refer to the ‘rLL’ (Left-Left) and ‘rLR’ (Left-Right) clusters as group A, and the ‘rR’ (Right) cluster as group B (S1 Fig). Note that these genomic clusters and the bioinformatic nomenclature thereof are distinct from classical clinical classifications of CRC samples that are based on physical location of the tumour. The validity of these genomic groups (or subtypes) were supported by multidimensional scaling (MDS) Fig 2, left; the adjacent normal samples (Fig 2, right) had a moderate degree of correspondence with the tumour-derived subgroups, but with no distinct groups. The biological relevance and robustness of these clusters were further supported by PARADIGM analysis––for which we used both transcriptomic and matched methylation data as input (S2 Fig)––where, with the exception of one sample (18T), the clusters were identical to those obtained by RPMM clustering of gene expression data (groups A and B).

In order to evaluate our CRC subtypes in the context of previously defined subtypes, we applied RPMM-based subtyping to a larger, well annotated CRC cohort (GSE13294, henceforth referred to as the Jorissen cohort [6]), of 155 colorectal adenocarcinomas, that had previously been subtyped by both De Sousa E Melo et al. and Sadamandam et al. [3,4]. Again three RPMM clusters were obtained, two of which were more closely related (rRL and rRR) and were combined for downstream analyses (referred to as group B, S3 Fig). MDS supported the RPMM-based clusters, which provided good separation of the data (S4 Fig). As shown in S3 Fig Our group B (84/155 samples) was predominantly composed of MSI+ samples (79% of MSI+ samples); in relation to previous classifications B group CRCs were dominated by the CCS2/CCS3 type of De Sousa E Melo et al. (65% CCS2/CCS3) and the inflammatory-, stem- and enterocyte-like samples of Sadanandam et al (40% of B group CRCs was composed of inflammatory-like samples (94% of which fell into the rRR subgroup of group B), 17% were stem-like samples, 12.5% were goblet-like, 24% were enterocyte-like and 7% were transit amplifying-like). Meanwhile, group A was dominated by CCS1 samples (72.5%) or the transit-amplifying type.

In our cohort, there were no significant differences in clinical characteristics between A and B-group patients; there was however a trend for increased cancers of the proximal colon, and for patients of White or Black ethnicity (as opposed to Mixed Ancestry) in B group patients (S1 Appendix).

*E*. *faecalis* colonisation was found to be significantly more frequent in B group CRCs (p = 0.05). Additionally, high levels of Fusobacterium (defined as the top quartile of Fusobacterium counts) were more common in B group CRCs (p = 0.06), and there was a trend to an increased frequency of high-level colonisation by any of the CRC-associated bacteria in B group CRCs (p = 0.1, Table 1).

**Table 1:** Comparison of bacterial colonisation between A and B group CRCs using Fisher’s exact test.

Feature	Group A (N = 9)	Group B (N = 10)	P (Fisher’s exact test)
FB-H	1	6	0.06
EF+ *	1	6	0.05
ETBF+	5	5	1
ClB+	3	3	1
afaC+	5	6	1
EPEC+	0	3	0.2
Colonisation-H (any)	5	13	0.1

*Two samples did not have data available.

FB-H: Fusobacterium-high; EF: *E*. *faecalis*; ETBF: Enterotoxigenic *B*. *fragilis*; ClB+: colibactin+ E. coli; EPEC: Enteropathogenic *E*. *coli*; Colonisation-H: frequency of high-level colonisations by any of the species tested.

### Increased CIMP in B group CRCs

Whole genome array-based methylation analysis was performed on each of 19 tumour samples, as described in S2 Appendix. CIMP status was predicted using an established array-based marker panel of CIMP, including *B3GAT2*, *FOXL2*, *KCNK13*, *RAB31*, and *SLIT1*, that was shown to identify CIMP+ (CIMP-H or CIMP-L) tumours with 100% sensitivity and 95.5% specificity, with 2.4% misclassification [5]. Interestingly, although the RPMM clustering used for subtyping did not take methylation data into consideration, B group CRCs were heavily enriched for CIMP+ status (80% CIMP+), S5 Fig.

### B group CRCs have inflammatory/goblet-like features

To assess the biological relevance of CRC subtypes in our cohort, we drew on the five subtypes reported by Sadanandam et al. 2013, that were linked to specific colonic crypt cell types based on gene expression profiles, the degrees of ‘stemness’ and Wnt signaling [3]. The five transcriptional CRC subtypes were: goblet-like, enterocyte, transit-amplifying, inflammatory or stem-like. Importantly, they found that 94% of the inflammatory subtype samples were MSI+, as opposed to 14% and 33% of transit-amplifying and stem-like subtypes, respectively [3]. Using the CRCassigner-786 signature of Sadanandam et al., each of the 786 genes in the panel were allocated to one of the five cell type-specific groups based on the maximum Prediction Analysis of Microarrays (PAM) score [3]. We next performed hierarchical clustering on each of these five sets of genes, in order to assess the degree of correspondence of our samples to each of the five subtypes; samples with increased gene expression relative to the rest of the cohort suggests increased correspondence to that subtype.

**Fig 3:**
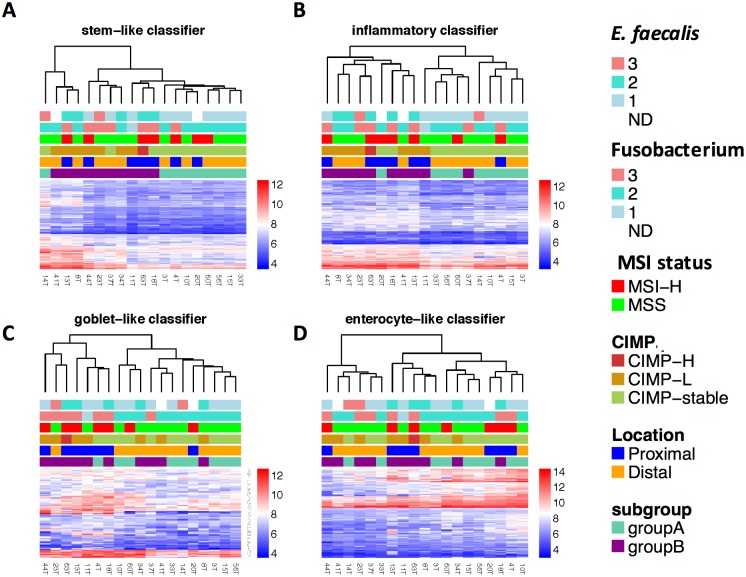
CRC classification according to the CRCassigner-786 classifier of Sadanandam et al. *E*. *faecalis* colonisation level category (1 = negative; 2 = low-level; 3 = high-level); *Fusobacterium* spp. colonisation level category (1 = negative; 2 = low-level; 3 = high-level). The legend categories on the right are presented in the same order as the row annotations at the top of the graph. The scale on the right represents log2 expression values.

For the transit amplifying-like panel of genes there was very little discernable difference between samples (result not shown). For the remaining four subtypes, subgroups were discernable from each of the clustering dendrograms, with varying degrees of correspondence with B group CRCs, Fig 3.

Strikingly, 9 of 10 B group CRCs (44T, 8T, 34T, 23T, 63T, 20T, 18T, 41T and 13T) clustered together based on the inflammatory subtype panel, with increased expression of several inflammation-related genes; this group included 8 of 10 CIMP+ samples and 5 of 7 MSI+ samples. Importantly, CRCs that had increased inflammatory and goblet-like gene expression signatures were enriched for high-level colonisation by *Fusobacterium* spp., with 6 of 7 of *Fusobacterium*-high infected samples present in the goblet-like cluster. Further, 6 of 7 *E*. *faecalis*+ samples fell in the inflammatory cluster (Fig 3). Seven samples (44T, 23T, 63T, 13T, 11T, 4T, 18T) had a relative increase in goblet-like expression, and most MSI+ samples (5 of 7) belonged to this cluster––interestingly these included both hereditary non-polyposis colorectal cancer (HNPCC) and sporadic MSI+ samples. Five of 7 goblet-like samples were also predicted to be CIMP+; moreover 6 of 7 were located in the proximal colon and these all belonged to the B group.

The transcriptomic-based inflammatory/goblet-like features of B group CRCs are supported by tumour biopsy pathology reports, where 5 of 9 B group patients for whom pathology reports were available displayed signs of inflammation and/or had a visible mucinous component. Two more patients in the inflammatory-subtype presented with diverticular disease and mucinous metaplasia of the appendix, respectively. In group A, only 1 of 8 patients for whom pathology reports were available had a reported mucinous component (4T), while another patient presented with diverticulae (10T).

Our results thus agree with the proposed merging of the Sadanamdam et al. goblet and inflammatory subtypes into one subtype (De Sousa E Melo et al. CCS2 subtype) [12], and demonstrate that many of these samples are MSI-H (5/7) and/or CIMP+.

### Altered gene expression and host signaling pathways in B group CRCs

Based on the finding that the B group of our cohort as well as the B group of the Jorissen cohort were dominated by MSI+ samples, with an increase in inflammatory gene expression, we next established the overlap in subtype-specific gene expression and pathway-level differences (group A vs. B) in our cohort with that of the Jorissen cohort. For each cohort, we performed a) differential gene expression analysis between groups A vs. B, followed by b) pathway analysis on the results from (a), and c) differential analysis of IPLs obtained from PARADIGM, separated by hierarchical clustering.

We found 4671 and 5571 genes at an absolute fold change cutoff of 1.25, and 296 and 546 genes at an absolute fold change cutoff of 2 in our cohort and in the Jorissen cohort, respectively (FDR ≤ 0.05). Of the 4671 genes differentially expressed in our cohort, 1619 overlapped with Jorissen subgroup comparison results, 78% (1266/1619) of which were consistently up- or down-regulated in both cohorts. Meanwhile, 19 genes were differentially altered in both cohorts at an absolute fold change ≥ 2 (FDR ≤ 0.05), 18 (95%) of which were consistently up- or down-regulated in the B group of both cohorts (Table 2). Nine of these have previously been linked to inflammation and/or IBD.

**Table 2:** Genes differentially expressed at an |FC| ≥ 2 and FDR ≤ 0.05 between subtypes in both cohorts.

Gene Symbol	GeneST (FDR)	GeneST (FC)	Jorissen (FDR)	Jorissen (FC)	consistent
C10orf99	3.3E-02	-2.5	4.3E-03	-2.4	yes
COL12A1	1.4E-03	3.6	4.8E-06	2.1	yes
CXCL10	4.9E-02	2.8	2.3E-06	2.8	yes
FCGR2A	1.0E-02	2.5	7.1E-14	2.4	yes
HSPA4L	1.6E-02	2.8	9.1E-11	2.6	yes
IL1B	7.0E-03	3.2	8.9E-09	2.7	yes
IL8	5.4E-03	4.1	6.1E-06	2.9	yes
MMP1	4.6E-02	3.0	2.7E-05	2.6	yes
MMP12	1.3E-03	4.4	5.0E-08	3.0	yes
NR4A2	1.5E-02	2.2	4.8E-06	2.1	yes
PKIB	4.1E-03	-2.2	7.7E-05	2.0	no
PLA2G4A	3.2E-02	2.4	1.9E-04	2.3	yes
PLK2	5.8E-04	2.0	3.8E-11	2.8	yes
POSTN	1.8E-02	3.7	2.1E-07	3.0	yes
PTGS2	2.6E-03	3.9	1.2E-08	3.0	yes
REG1A	3.3E-02	6.2	3.2E-02	2.5	yes
TDO2	4.1E-02	3.2	7.5E-06	2.0	yes
TNFAIP6	3.7E-03	3.0	5.1E-09	2.3	yes
VCAN	2.1E-02	2.4	7.1E-08	2.3	yes

For Ingenuity Pathway Analysis (IPA) genes significantly altered between subtypes (FDR ≤ 0.05, FC ≥ 1.25) were used to investigate a) canonical pathways b) upstream regulators and c) diseases and functions that defined CRC subtypes. We then determined the overlap between pathways significantly altered in both cohorts.

Fifty-four and 96 canonical pathways were significantly overrepresented (p ≤ 0.05) in B group CRCs in our cohort and in the Jorissen cohort, respectively (S1 Appendix). Fifteen canonical pathways were significantly overrepresented in B vs. A group CRCs in both cohorts, including DNA and protein damage response, and cell cycle regulation pathways (Table 3).

**Table 3:** Canonical pathways predicted to be significantly altered in both cohorts in B vs. A group samples. The first three data columns refer to our cohort, and the last three to the Jorissen cohort.

Ingenuity Canonical Pathways	log(p-value)	Down	Up	log(p-value) (Jorissen)	Down (Jorissen)	Up (Jorissen)
Role of BRCA1 in DNA Damage Response	10.6	0/60 (0%)	46/60 (77%)	1.85	12/61 (20%)	17/61 (28%)
Protein Ubiquitination Pathway	9.27	20/249 (8%)	126/249 (51%)	6.54	21/251 (8%)	101/251 (40%)
Cell Cycle: G2/M DNA Damage Checkpoint Regulation	8.48	1/48 (2%)	32/48 (67%)	1.79	7/49 (14%)	17/49 (35%)
Hereditary Breast Cancer Signaling	7.29	7/111 (6%)	61/111 (55%)	1.68	16/112 (14%)	32/112 (29%)
Cell Cycle: G1/S Checkpoint Regulation	4.86	7/63 (11%)	32/63 (51%)	1.38	12/63 (19%)	16/63 (25%)
Regulation of eIF4 and p70S6K Signaling	4.54	10/141 (7%)	72/141 (51%)	1.42	13/140 (9%)	44/140 (31%)
Cyclins and Cell Cycle Regulation	4.46	10/77 (13%)	36/77 (47%)	1.31	11/77 (14%)	22/77 (29%)
DNA damage-induced 14-3-3“€ Signaling	3.47	3/19 (16%)	11/19 (58%)	1.63	2/19 (11%)	9/19 (47%)
Gluconeogenesis I	2.83	2/24 (8%)	12/24 (50%)	1.33	2/23 (9%)	10/23 (43%)
mTOR Signaling	2.19	16/178 (9%)	73/178 (41%)	1.42	21/181 (12%)	51/181 (28%)
Polyamine Regulation in Colon Cancer	2.12	1/22 (5%)	12/22 (55%)	1.68	0/21 (0%)	12/21 (57%)
dTMP De Novo Biosynthesis	1.86	0/5 (0%)	4/5 (80%)	1.35	0/5 (0%)	4/5 (80%)
Androgen Signaling	1.44	11/109 (10%)	42/109 (39%)	1.84	13/110 (12%)	35/110 (32%)
Calcium Transport I	1.37	3/9 (33%)	2/9 (22%)	1.38	2/9 (22%)	4/9 (44%)
Endoplasmic Reticulum Stress Pathway	1.34	0/21 (0%)	12/21 (57%)	1.68	2/21 (10%)	10/21 (48%)

Sixty-eight IPA-predicted upstream regulators were differentially activated by subgroup in both cohorts (S1 Appendix), 99% of which had consistent direction of predicted activation state.

Regarding IPA diseases and functions, the 20 highest scoring diseases and functions by p-value (threshold p ≤ 0.05, |activation z-score| ≥ 2) included *DNA Replication*, *Recombination*, *Repair*, *Cell Cycle* and *Infectious Disease* categories in our cohort (S1 Appendix) and *Cellular Growth and Proliferation*, *Infectious Disease*, and *Cancer*, and functions related to tumour progression and metastasis in the Jorissen cohort (S1 Appendix). Eleven diseases and functions overlapped between the two cohorts, and intriguingly, six of these fell into the *Infectious Disease* category (S1 Appendix). Interestingly, increased *Viral Infection* was indicated in B group CRCs of both cohorts as well as decreased *Bacterial Infection* in the Jorissen cohort (p = 2e-10, z-score = -3). Heatmaps of the genes in these pathways show a general increase in genes implicated in *Viral Infection* (which is predicted to be increased in group B) and also in *Bacterial Infection* (which is predicted to be decreased in group B) in B group CRCs (S6a and S6b Fig). The *Viral* and *Bacterial Infection* functions were based on 912 and 218 genes, respectively (with 124 shared between the two categories) that were differentially expressed between B and A group CRCs of the Jorissen cohort. Additionally, *cellular proliferation* and *cell viability*, as well as *metabolism and transport of proteins* were predicted to be increased in B group CRCs of both cohorts.

PARADIGM analyses (using transcriptomic and methylome data for our cohort and transcriptome data only for the Jorissen cohort) were in agreement with the IPA results: 1464 and 3619 PARADIGM integrated pathway levels (IPLs) differentially activated (FDR ≤ 0.05, absolute difference in group medians ≥ 0.25) between B and A group CRCs, in our cohort and in the Jorissen cohort, respectively. Of these, 570 IPLs were common to both cohorts, 499 (88%) of which had consistent direction of activation. Shared abstract processes included DNA damage response-related pathways, *activation of caspase activity by cytochrome c* and *prostaglandin biosynthesis* (S1 Appendix).

### The biological response seen in B group CRCs is not MSI-specific

Compared to their microsatellite-stable counterparts, MSI+ CRCs have a heightened immune response that is evident macroscopically and at the molecular level [13]. This tumour-specific immune response is caused by antigenic MSI-induced frameshift mRNAs and/or peptides [14,15]. We therefore investigated the putative role of MSI in B-group-specific alterations in immune-related pathways, by comparing the level of activation of IPA canonical pathways related to inflammation, infection and oxidative stress in relation to MSI status vs. CRC subtype. The results (S1 Appendix) clearly demonstrate stronger evidence for upregulation of these pathways in B group CRCs (of which 21% are MSS) as opposed to MSI+ CRCs, which suggests that this effect is unlikely to be driven by MSI in itself.

An important consequence of the anti-tumoural immune response in MSI+ CRCs is selective pressure towards immune evasion [15]. Mechanisms that contribute towards immune evasion in MSI+ CRCs include alteration in antigen-presentation machinery, specifically in HLA class I-mediated antigen presentation that can be compromised by mutations in *B2M* (30–60% of MSI+ CRCs), or through loss or downregulation of HLA class I heavy chains (~60% of MSI+ CRCs) [15]. Further, alterations in antigen processing machinery may also facilitate immune evasion [15]. *HLA* gene expression was therefore compared between a) MSI vs. MSS cancers, and b) B vs. A group CRCs in the Jorissen cohort. Strikingly, multiple *HLA* class II (*HLA-D*) genes were preferentially upregulated in B group CRCs, while no *HLA* class I genes were differentially expressed in either comparison (S1 Appendix). These results suggest that induction of MHC class II antigen presentation occurs in B group CRCs (likely by tumour infiltrating lymphocytes), and that these antigens are not specific to MSI+ cancers. These findings provide further support for the presence of foreign antigens in B group CRCs, which may be of microbial origin.

### E. faecalis- and Fusobacterium-specific host gene expression and pathway-level changes

Host gene expression was found to be significantly altered in *E*. *faecalis*-colonised CRCs where 128 genes were differentially expressed in *E*. *faecalis*-positive vs. -negative CRCs (FDR ≤ 0.05, FC ≥ 2), S1 Appendix. In CRCs colonised by Fusobacterium-H, only three genes were significantly upregulated, all three belonging to the regenerating islet-derived family of genes: *REG3A* (FDR = 0.0023, FC = 15.4), *REG1A* (FDR = 0.0072, FC = 22.8), *REG1P* (FDR = 0.012, FC = 6.7). We did not find differentially expressed genes for the other bacteria included in this study, nor for the comparisons made in normal tissue samples (S1 Appendix); it should however be noted that for some of the comparisons the small numbers in some of the groups could have precluded identification of differentially expressed genes.

Based on pathway analysis, several pathways related to cancer metastasis and invasion were predicted to be activated in *E*. *faecalis*-colonised CRCs, including the IPA Diseases and Functions Annotations: *proliferation of cells*; *metastasis*; *invasion of tumour cell lines*; *epithelial-mesenchymal transition*; and *cell movement of colorectal cancer cell lines* (Table 4). The top-scoring canonical pathways were *Antigen Presentation Pathway*, followed by *OX40 Signaling Pathway*, *Growth Hormone Signaling* and *Colorectal Cancer Metastasis Signaling* (S1 Appendix).

**Table 4:** Diseases and functions activated in *E*. *faecalis*-colonised CRCs (p≤0.05, |z-score| ≥ 2). Boldface entries were also significant in the comparison between B vs. A group CRCs.

Categories	Diseases or Functions Annotation	p-Value	Activation z-score
**Cellular Growth and Proliferation**	**proliferation of cells**	**1.70E-04**	**3.9**
Cellular Development, Skeletal and Muscular System Development and Function, Tissue Development	differentiation of smooth muscle cells	3.90E-04	2.2
Cancer	metastasis	9.50E-04	3.1
Cellular Movement	invasion of tumour cell lines	9.70E-04	2.9
Connective Tissue Disorders, Developmental Disorder, Skeletal and Muscular Disorders	craniofacial abnormality	2.80E-03	-2.4
Cellular Movement	invasion of cells	6.90E-03	2.8
Cellular Development	epithelial-mesenchymal transition	7.00E-03	2.6
Cellular Movement	invasion of breast cancer cell lines	7.40E-03	2.1
Cardiovascular System Development and Function	neovascularization	8.60E-03	2
Cancer	neoplasia of cells	1.10E-02	2.2
Organismal Development	size of body	1.30E-02	3.9
Inflammatory Disease, Respiratory Disease	pulmonary emphysema	1.50E-02	-2.2
**Cellular Development**	**epithelial-mesenchymal transition of tumour cell lines**	**1.50E-02**	**2.3**
Developmental Disorder, Immunological Disease	hypoplasia of thymus gland	1.80E-02	-2.6
Cellular Movement	cell movement of colon cancer cell lines	1.80E-02	2.6
Cellular Movement	migration of colon cancer cell lines	1.90E-02	2.4
Cellular Development, Skeletal and Muscular System Development and Function, Tissue Development	differentiation of muscle cells	2.10E-02	2.2
Cell-To-Cell Signaling and Interaction, Tissue Development	adhesion of epithelial cells	2.30E-02	2

## Discussion

Microbial origins of numerous cancers are well established today in, for example, gastric and cervical cancers, and it seems likely that others will follow. Altered levels of specific pathogenic bacteria have been reported in patients with IBD and CRC, but causality in these diseases remains unproven. Here, we have identified specific host gene expression and pathway alterations in *E*. *faecalis* and Fusobacterium-H colonised CRCs that provide new evidence for plausible mechanistic links between bacterial colonisation and the development and progression of a specific genomic subtype of CRC. Together with the relative increase in inflammatory and DNA damage pathways found in this specific CRC subtype, our data suggests that polymicrobial colonisation of the colonic epithelium may be an important aspect of colonic tumourigenesis in certain CRCs.

*E*. *faecalis* has previously been reported to be found at significantly higher levels in stool samples from CRC patients compared to healthy controls [16], and its oncogenic potential has been suggested based on its production of extracellular superoxide, which leads to inflammation, DNA damage and CRC in IL-10 knockout mice [17–19]. *E*. *faecalis* can also induce aneuploidy and tetraploidy in vitro [17]. However, we are the first to report *E*. *faecalis* in association with a specific CRC subtype. We further show that CRC invasion- and metastasis-related genes and pathways were significantly upregulated in *E*. *faecalis*+ CRCs, including the canonical pathway *CRC Metastasis Signaling* and the diseases and functions categories *metastasis*, *cell movement of colon cancer cell lines* and *migration of colon cancer cell lines* in. Together these results imply a more aggressive phenotype for *E*. *faecalis*+ CRCs.

Although *E*. *faecalis* is a normal constituent of the human microbiome, it is also a common source of infection, a disparity most likely explained by strain-specific virulence factors, including lipoteichoic acid, AS and bacteriocin [20]. These virulence factors can induce inflammatory cytokines (including TNF-β, IFN-γ and TNF-α) [20] that may be relevant in the pathogenesis of *E*. *faecalis*+ CRCs. Indeed, in our study, TNF and IFN-γ were predicted by pathway analysis to be activated in *E*. *faecalis*+ CRCs, consistent with mouse models [21]. Furthermore, we observed that *CXCL10* was upregulated in *E*. *faecalis*+ CRCs (FDR = 0.009, FC = 5.8), consistent with its reported upregulation in *Enterococcus*-administered IL-10^-/-^ mice [21,22]. In addition, *BMI1* polycomb ring finger oncogene—an intestinal stem cell marker that is overexpressed in various cancers [23]–was upregulated two-fold in *E*. *faecalis*+ CRCs, which is striking since high expression of BMI1 is significantly associated with metastasis [24,25] and poor survival [26] in CRC patients. Importantly, aberrant BMI1 expression has been found in premalignant gastrointestinal lesions [23], which points to its possible role in cancer initiation. Taken together, these results suggest that *E*. *faecalis*-dependent regulation of specific host genes is likely to be involved in disease progression in B group CRCs.

In determining the pathway features that distinguish CRC subtypes, we found that DNA and protein damage response-related processes were significantly increased in B group CRCs, which is relevant since *E*. *faecalis* is known to be able to induce DNA damage through the production of ROS; we therefore speculate that *E*. *faecalis* underlies the pathway-level DNA and protein damage responses seen in the majority of B group CRCs. This suggestion is supported by the findings by Barnett et al. who reported Enterococcus-specific alterations in the *Cell Cycle*: *G2/M DNA Damage Checkpoint Regulation* pathway in IL10-/- mice [21], and by the fact that oxidant-generating enzymes—including NOX (NADPH oxidase) and DUOX (Dual oxidase) enzymes, myeloperoxidase (MPO) and inducible nitric oxide synthase (iNOS)—were not upregulated in B group CRCs at the mRNA level. In fact, we found to the contrary that both *NOX1* and *NOXA1* were significantly downregulated in B group cancers (NOX1, FC = –2.3, Jorissen cohort; NOXA1, FC = 1.3, both cohorts), which further supports the idea that exogenous, bacterially-derived ROS is a driver in B group CRCs.

Fusobacterium spp., and in particular *F*. *nucleatum*, are more commonly found in CRC patients compared with healthy controls, with a marked increase in the colonisation of tumour compared to adjacent normal mucosal tissue [27–29]. In our cohort, Fusobacterium occurred at significantly higher levels in tumour samples (p = 0.0003) [2], even before taking subtypes into consideration. *F*. *nucleatum* can act as a scaffold for secondary bacterial colonisers, resulting in a structured biofilm [30–32], whilst *E*. *faecalis* has been reported to co-aggregate with *F*. *nucleatum* in certain infections [33], implying that Fusobacterium may facilitate colonisation of other potentially oncogenic pathogens in the colon. This is supported by co-occurrence network analysis of metagenomic signatures, which identified a subset of microbes significantly associated with *F*. *nucleatum* in CRC biopsies [32,34]. Interestingly, *F*. *nucleatum* can adapt to oxidative stress [35,36] and exhibits enhanced pathogenicity in mice under these conditions [37,38], implying potential dual roles in CRC pathogenesis.

An important novel finding from our data is that REG-family gene expression is significantly elevated in Fusobacterium-H CRCs with dramatic fold change increases detected; this includes *REG1A*, *REG3A* and *REG1P*, as well as a borderline significant expression for *REG1B* (FC = 12.2, FDR = 0.17). REG proteins are members of the C-type lectin superfamily and have important roles in proliferation and differentiation in a range of cell types. Of the REGs, only REG4 is constitutively expressed in the colon but several REG proteins are aberrantly expressed in inflammatory pathologies including IBD, where REG1A, REG1B and REG3A are all expressed at the intestinal crypt base by metastatic Paneth cells [39]; REG1A and REG1B have also been reported to be concomitantly upregulated in CRC [40], but no mechanistic rationale has been provided previously. Notably, REG1A is a downstream target of Wnt pathway activation [41] and its expression has been reported to be induced through the induction of IL-8 [42]. It is therefore relevant that here we observed an increase in inflammatory IL-8 signaling in B group CRCs, consistent with previous findings that Fusobacterium spp. are associated with increased IL-8 *in vitro* [43] and *in vivo* [34,44]. It therefore seems reasonable to suppose that the upregulation of REG genes found in our study may be directly caused by increased colonisation of the tumour interface by pro-inflammatory Fusobacterium. Interestingly, REG1A is associated with poor prognosis in CRC [45], and recurrence and/or distant metastasis and short median survival in patients with MSI+ tumours [46]; it is noteworthy therefore that the upregulation of REG1A and REG3A seen here is specific to Fusobacterium-high tumours, rather than MSI+ tumours, with these genes being not significantly differentially expressed in MSI+ vs. MSI- tumours in our cohort.

Taken together, our results point strongly towards a mechanistic link between *E*. *faecalis* and Fusobacterium-H colonisation and the biological responses seen in B group CRCs. Moreover, our pathway level results imply a possible affect of *E*. *faecalis* and Fusobacterium-H on CRC progression/metastasis, although further studies with animal models will be required to investigate this in more detail and to establish possible causality.

One feature of B group CRCs with potentially important clinical relevance is the significant upregulation of *COX-2 (PTGS2)*, which implies that aspirin (a *COX-2* inhibitor) might be useful in preventing B group CRCs by blocking inflammation—an idea supported by our pathway analyses, which list aspirin as a ‘deactivated’ upstream regulator (z-score –4.4 p-val 9.6e-8; Jorissen cohort). It is interesting in this regard that regular prophylactic aspirin use specifically reduces the risk of developing CRCs that overexpress *COX-2* [47]. The level of protection conferred by aspirin on a CRC subtype-specific basis therefore warrants further investigation, especially since recent studies offer compelling evidence for the use of aspirin as an adjuvant therapy for CRC [48]. Our findings should therefore have significant implications for future studies on microbially-associated CRCs and may also have diagnostic and therapeutic implications for specific colorectal cancer subtypes.

## Supporting Information

S1 Appendix Supplementary tables. (PDF)

S2 Appendix Supplementary information, including array-based methylation and CIMP analysis; and PARADIGM pathway analysis. (PDF)

S1 Fig RPMM-based clustering of the top quartile (N = 8325) most variable transcript clusters. Levels of bacterial colonisation (described in the Methods section) are indicated on the figure legend, where 3: high- level colonisation, 2: low-level colonisation, 1: no colonisation. ETBF: Enterotoxigenic *Bacteroides fragilis*; ClB: *ClB*/*pks*+ *E*. *coli*, FB: *Fusobacterium* spp., afaC: *afaC*+ *E*. *coli*; EF: *Enterococcus faecalis*. The legend categories on the right are presented in the same order as the row annotations at the top of the graph. The scale on the right represents log2 expression values.
(PDF)

S2 Fig Hierarchical clustering of the 5334 most variable PARADIGM IPLs. Two main clusters can be distinguished that are identical to the RPMM gene-expression clusters except for 18T. The scale on the right represents row-scaled expression values. EF.cat: *E*. *faeclis* colonisation category (1 = negative; 2 = low-level; 3 = high- level); ETBF.cat: ETBF colonisation category (1 = negative; 2 = low-level; 3 = high-level); ClB.cat: pks+ *E*. *coli* (1 = negative; 2 = low-level; 3 = high-level); EPEC.cat (1 = negative; 2 = positive); FB.cat: Fusobacterium colonisation category (1 = negative; 2 = low-level; 3 = high-level); afaC.cat: afaC+ *E*. *coli* (1 = negative; 2 = low-level; 3 = high-level); ND: not determined.
(PDF)

S3 Fig RPMM-based clustering of the most variable quartile of transcript clusters (N = 13669) for the Jorissen cohort (N = 155). RPMM clusters are displayed alongside the previously established de Sousa and Sadanandam subtypes and MSI-status for each sample. Here, only the top 1000 most variable probes are displayed, although clustering was conducted on the top quartile most variable probes. The legend categories on the right are presented in the same order as the row annotations at the top of the graph. The scale on the right represents log2 expression values. The rRL and rRR clusters together are referred to as group B, while the rL cluster is referred to as group A.
(PDF)

S4 Fig Multidimensional scaling of the top quartile (N = 13669) most variable transcript clusters used for RPMM clustering of the Jorissen cohort. The three figures are identical apart from the annotation colours used, where samples have been coloured by the De Sousa E Melo (left), Sadanandam (middle) or RPMM (right) subgroups.
(PDF)

S5 Fig Predicting CIMP-status using an array-based marker panel. RPMM-based clustering of probes mapping to CpG islands in the Hinoue CIMP marker panel (*B3GAT2*, *FOXL2*, *KCNK13*, *RAB31*, and *SLIT1*). Samples in the rL cluster are considered to be CIMP+. The legend categories on the right are in the same order as the row annotations at the top of the graph. The scale on the right of the heatmap indicates beta values (0–1). Patients 13, 18, 20 and 4 were diagnosed with HNPCC.
(PDF)

S6 Fig **a)** Hierarchical clustering of the 218 genes differentially expressed between groups A and B that were classified under the IPA diseases and functions category *Bacterial Infection*; **b)** Hierarchical clustering of the 912 genes differentially expressed between groups A and B that were classified under the IPA diseases and functions category *Viral Infection*.
(TIFF)
