# Supplementary material for: Bacterially-Associated Transcriptional Remodelling in a Distinct Genomic Subtype of Colorectal Cancer Provides a Plausible Molecular Basis for Disease Development
Source: PLoS One. 2016 Nov 15;11(11):e0166282. doi: 10.1371/journal.pone.0166282 (PMC5112903; doi:10.1371/journal.pone.0166282)
Supplement: S1 Appendix — (PDF) [file pone.0166282.s001.pdf]

Supplemental Table 1a: Participant-level characteristics. FF = fresh-frozen; Tissue type: T=tumour tissue; Ethnicity: MA=mixed ancestry, C = caucasian, B = black, I=indian; Gender: M=male, F=female; RT=radiotherapy; N=No RT received before surgery; Stage: Dukes stage of tumour tissue.

| **Sample** | **Specimen** | **Tumour** | **Tissue** | **Patient** | **Age** | **Ethnicity** | **RT** | **Gender** | **BMI** | **Stage** | **Location** | **Site** |
|---|---|---|---|---|---|---|---|---|---|---|---|---|
| 10T | FF | Sporadic | T | 10 | 63 | MA | N | M | 24.7 | III | Distal | Descending colon |
| 11T | FF | Sporadic | T | 11 | 84 | C | N | M | 28.7 | I | Proximal | Transverse colon |
| 13T | FF | HNPCC | T | 13 | 46 | MA | N | F | 22.3 | II | Proximal | Caecum |
| 14T | FF | Sporadic | T | 14 | 80 | MA | N | M | 23.5 | IV | Distal | Sigmoid colon |
| 15T | FF | Sporadic | T | 15 | 74 | I | N | M | 24.2 | II | Distal | Rectum |
| 18T | FF | HNPCC | T | 18 | 44 | MA | N | F | NA | I | Proximal | Transverse colon |
| 20T | FF | HNPCC | T | 20 | NA | MA | N | F | NA | NA | Proximal | Caecum |
| 23T | FF | Sporadic | T | 23 | 70 | MA | N | F | NA | III | Distal | Splenic flexure |
| 33T | FF | Sporadic | T | 33 | 69 | MA | N | F | 28.7 | III | Distal | Rectum |
| 34T | FF | Sporadic | T | 34 | 70 | MA | N | F | 25.4 | III | Distal | Rectum |
| 37T | FF | Sporadic | T | 37 | 49 | B | N | M | 20 | III | Distal | Proximal descending |
| 3T | FF | Sporadic | T | 3 | 70 | MA | N | M | 29.5 | III | Distal | RSJ |
| 41T | FF | Sporadic | T | 41 | 71 | MA | N | F | 31.6 | II | Distal | Rectum |
| 44T | FF | Sporadic | T | 44 | 36 | B | N | M | 18.2 | III | Proximal | Caecum |
| 48T | FF | Sporadic | T | 48 | 37 | MA | N | M | 30.7 | IV | Distal | NA |
| 4T | FF | HNPCC | T | 4 | 44 | MA | N | F | 26.7 | III | Proximal | Caecum |
| 55T | FF | Sporadic | T | 55 | 64 | MA | N | M | 25.8 | III | Distal | NA |
| 56T | FF | Sporadic | T | 56 | 54 | MA | N | F | 26.9 | III | Distal | RSJ |
| 60T | FF | Sporadic | T | 60 | 65 | MA | N | M | NA | II | Distal | RSJ |
| 63T | FF | Sporadic | T | 63 | 78 | C | N | F | 26.8 | II | Proximal | Hepatic flexure |

Supplemental Table 1b. Bacterial copy numbers detected (per 50ng of DNA extracted from tissue). ETBF: Enterotoxigenic *B. fragilis*; EPEC=Enteropathogenic *E. coli;* EF=E. faecalis; FB=Fusobacterium; afaC=afaC gene from AIEC; ClB=Colibactin gene from AIEC.

| **Sample** | **ETBF** | **EPEC** | **EF** | **FB** | **afaC** | **ClB** |
|---|---|---|---|---|---|---|
| **10N** | **12** | **0** | **NA** | **8** | **2177** | **0** |
| **10T** | **139** | **0** | **0** | **1** | **170** | **0** |
| **11N** | **0** | **0** | **0** | **0** | **0** | **0** |
| **11T** | **0** | **0** | **0** | **0** | **0** | **0** |
| **13N** | **374** | **0** | **0** | **65** | **3787** | **0** |
| **13T** | **3186** | **0** | **1** | **3273** | **19200** | **0** |
| **14N** | **3423** | **0** | **331** | **67** | **261** | **0** |
| **14T** | **987** | **0** | **151** | **271** | **729** | **0** |
| **15N** | **2** | **0** | **0** | **9** | **4787** | **1172** |
| **15T** | **0** | **0** | **0** | **17** | **9887** | **1707** |
| **18N** | **0** | **0** | **5** | **377** | **1937** | **0** |
| **18T** | **0** | **0** | **3** | **2610** | **7593** | **994** |
| **20N** | **3** | **0** | **NA** | **0** | **0** | **0** |
| **20T** | **13** | **0** | **NA** | **2** | **0** | **0** |
| **23N** | **5** | **0** | **1** | **26** | **0** | **0** |
| **23T** | **3** | **0** | **250** | **4820** | **0** | **0** |
| **33N** | **0** | **0** | **0** | **3** | **0** | **677** |
| **33T** | **0** | **0** | **0** | **14** | **0** | **622** |
| **34N** | **0** | **52** | **0** | **25** | **30900** | **1263** |
| **34T** | **3** | **63** | **1** | **289** | **33433** | **1400** |
| **37N** | **1620** | **0** | **NA** | **1897** | **0** | **3693** |
| **37T** | **35300** | **0** | **0** | **4773** | **0** | **1800** |
| **3N** | **0** | **0** | **0** | **1** | **0** | **0** |
| **3T** | **0** | **0** | **0** | **3** | **0** | **0** |
| **41N** | **0** | **0** | **NA** | **69** | **0** | **0** |
| **41T** | **0** | **0** | **NA** | **213** | **2** | **0** |
| **44N** | **0** | **3037** | **0** | **1110** | **1197** | **36** |
| **44T** | **0** | **1111** | **0** | **68700** | **156** | **0** |
| **48N** | **2328** | **0** | **0** | **1** | **0** | **0** |
| **48T** | **1106** | **0** | **0** | **44** | **0** | **0** |
| **4N** | **0** | **0** | **0** | **20** | **195** | **0** |
| **4T** | **3** | **0** | **0** | **60767** | **6537** | **0** |
| **55N** | **4** | **0** | **0** | **99** | **0** | **5777** |
| **55T** | **3** | **0** | **0** | **9** | **0** | **536** |
| **56N** | **698** | **0** | **0** | **46** | **0** | **0** |
| **56T** | **648** | **0** | **0** | **378** | **0** | **0** |
| **60N** | **20** | **0** | **0** | **3** | **2750** | **78** |
| **60T** | **0** | **0** | **0** | **22** | **5237** | **69** |
| **63N** | **0** | **0** | **NA** | **73** | **0** | **0** |
| **63T** | **0** | **13** | **4** | **2730** | **0** | **0** |

Supplemental Table 2. Summary of differential gene expression analyses conducted for ***tumour*** samples showing the number of transcript clusters differentially expressed for each comparison made.

| Model specified | Number of samples/category | FDR ≤ 0.05, FC ≥ 2 |
|---|---|---|
| EF+ vs. EF– | 7 vs. 10 | 128 |
| FB-H vs. FB-L/N | 7 vs. 12 | 3 |
| afaC-H vs. afaC-L/N | 5 vs. 14 | 0 |
| afaC+ vs. afaC– | 13 vs. 8 | 0 |
| ClB+ vs. ClB– | 6 vs.13 | 0 |
| ETBF-H vs. ETBF L/N | 3 vs. 16 | 0 |
| ETBF+ vs. ETBF– | 10 vs. 9 | 0 |
| EPEC+ vs. EPEC– | 3 vs. 16 | 0 |

H: high-level infection; L: low-level infection; N: no infection; FB: Fusobacterium; EF: *E. faecalis*

Supplemental Table 3: Summary of differential gene expression analyses conducted for ***normal*** samples showing the number of transcript clusters differentially expressed for each comparison made.

| Model specified | Number of samples/category | FDR ≤ 0.05 & FC ≥ 2 |
|---|---|---|
| ETBF+ vs. ETBF- | 10 vs. 9 | 0 |
| ETBF-H vs. ETBF-L/N | 4 vs. 20 | 0 |
| ClB+ vs. ClB- | 6 vs. 13 | 0 |
| afaC+ vs. afaC- | 10 vs. 9 | 0 |
| afaC-H vs. afaC-L/N | 3 vs. 16 | 0 |
| EF+ vs. EF- | 4 vs. 10 | 0 |
| FB-H vs. FB-L/N | 3 vs. 16 | 0 |
| FB+ vs. FB- | 16 vs. 3 | 0 |

H: high-level infection; L: low-level infection; N: no infection; FB: Fusobacterium; EF: *E. faecalis*

Supplemental Table 4: Descriptive characteristics by CRC subtype.

| | Group A (N = 9) | Group B (N = 10) | P (Fisher's exact) |
|---|---|---|---|
| **MSI-H** | 3 | 4 | 1.00 |
| **HNPCC+** | 2 | 2 | 1.00 |
| **Stage*** | | | |
| *I/II* | 2 | 5 | 0.37 |
| *III/IV* | 6 | 5 | 0.37 |
| **Site** | | | |
| *Proximal colon* | 2 | 5 | 0.35 |
| *Distal colon* | 5 | 2 | 0.35 |
| *Rectum* | 2 | 3 | 1.00 |
| **Age*** | | | |
| *≤ 60* | 2 | 4 | 0.63 |
| *> 60* | 6 | 6 | 1 |
| **Gender** | | | |
| *Male* | 5 | 4 | 0.66 |
| *Female* | 4 | 6 | 0.66 |
| **Ethnicity** | | | |
| *Mixed ancestry* | 8 | 5 | 0.14 |
| *Black* | 0 | 2 | 0.47 |
| *White* | 0 | 3 | 0.2 |
| *Indian* | 1 | 0 | 0.47 |

*One case had missing information

Supplemental Table 5a: Top 20 most significant IPA canonical pathways in group B vs. group A samples of our cohort.

| Ingenuity Canonical Pathways | -log(p- | Downregulat | Upregulated |
|---|---|---|---|
| EIF2 Signaling | 13.4 | 8/169 (5%) | 109/169 (64%) |
| Role of BRCA1 in DNA Damage Response | 10.6 | 0/60 (0%) | 46/60 (77%) |
| Protein Ubiquitination Pathway | 9.27 | 20/249 (8%) | 126/249 (51%) |
| Cell Cycle Control of Chromosomal Replication | 9.25 | 1/27 (4%) | 25/27 (93%) |
| Cell Cycle: G2/M DNA Damage Checkpoint Regulation | 8.48 | 1/48 (2%) | 32/48 (67%) |
| Mitotic Roles of Polo-Like Kinase | 8.32 | 4/63 (6%) | 39/63 (62%) |
| Hereditary Breast Cancer Signaling | 7.29 | 7/111 (6%) | 61/111 (55%) |
| Role of CHK Proteins in Cell Cycle Checkpoint Control | 6.52 | 4/55 (7%) | 36/55 (65%) |
| tRNA Charging | 5.72 | 0/38 (0%) | 26/38 (68%) |
| RAN Signaling | 5.61 | 0/16 (0%) | 15/16 (94%) |
| Mismatch Repair in Eukaryotes | 5.61 | 0/16 (0%) | 13/16 (81%) |
| Cell Cycle: G1/S Checkpoint Regulation | 4.86 | 7/63 (11%) | 32/63 (51%) |
| Regulation of eIF4 and p70S6K Signaling | 4.54 | 10/141 (7%) | 72/141 (51%) |
| Cyclins and Cell Cycle Regulation | 4.46 | 10/77 (13%) | 36/77 (47%) |
| ATM Signaling | 4.09 | 2/59 (3%) | 31/59 (53%) |
| Telomere Extension by Telomerase | 4.09 | 0/15 (0%) | 14/15 (93%) |
| Purine Nucleotides De Novo Biosynthesis II | 4.03 | 1/11 (9%) | 8/11 (73%) |
| DNA Double-Strand Break Repair by Homologous Recombination | 3.60 | 0/14 (0%) | 10/14 (71%) |
| DNA Double-Strand Break Repair by Non-Homologous End Joining | 3.60 | 0/14 (0%) | 11/14 (79%) |
| DNA damage-induced 14-3-3“€ Signaling | 3.47 | 3/19 (16%) | 11/19 (58%) |
| Estrogen-mediated S-phase Entry | 3.45 | 3/24 (13%) | 16/24 (67%) |

Supplemental Table 5b: Top 20 most significant IPA canonical pathways in group B vs. group A samples of the Jorissen cohort.

| Ingenuity Canonical Pathways | -log(p-value) | Downregulated | Upregulated |
|---|---|---|---|
| Antigen Presentation Pathway | 9.28 | 1/34 (3%) | 28/34 (82%) |
| IGF-1 Signaling | 8.40 | 14/96 (15%) | 46/96 (48%) |
| Glucocorticoid Receptor Signaling | 8.38 | 24/256 (9%) | 106/256 (41%) |
| Role of NFAT in Regulation of the Immune Response | 8.31 | 14/158 (9%) | 74/158 (47%) |
| CD28 Signaling in T Helper Cells | 7.83 | 9/107 (8%) | 55/107 (51%) |
| OX40 Signaling Pathway | 7.71 | 2/46 (4%) | 32/46 (70%) |
| Role of Tissue Factor in Cancer | 7.35 | 11/107 (10%) | 52/107 (49%) |
| Cdc42 Signaling | 7.21 | 8/121 (7%) | 61/121 (50%) |
| B Cell Receptor Signaling | 7.17 | 16/167 (10%) | 73/167 (44%) |
| Leukocyte Extravasation Signaling | 6.76 | 13/191 (7%) | 85/191 (45%) |
| Integrin Signaling | 6.68 | 18/194 (9%) | 81/194 (42%) |
| Protein Ubiquitination Pathway | 6.54 | 21/251 (8%) | 101/251 (40%) |
| Virus Entry via Endocytic Pathways | 6.51 | 10/89 (11%) | 43/89 (48%) |
| Caveolar-mediated Endocytosis Signaling | 6.19 | 6/71 (8%) | 38/71 (54%) |
| HGF Signaling | 6.16 | 12/104 (12%) | 47/104 (45%) |
| PI3K/AKT Signaling | 6.14 | 13/120 (11%) | 53/120 (44%) |
| IL-17 Signaling | 5.94 | 4/72 (6%) | 40/72 (56%) |
| Type I Diabetes Mellitus Signaling | 5.63 | 3/100 (3%) | 53/100 (53%) |
| Prostate Cancer Signaling | 5.61 | 7/80 (9%) | 40/80 (50%) |
| PKCëü Signaling in T Lymphocytes | 5.60 | 7/107 (7%) | 52/107 (49%) |

Supplemental Table 6a: Top 20 most significant upstream regulators predicted to be significantly altered in group B vs. group A samples in **our cohort**, with absolute activation z-scores ≥ 2. Chemical upstream regulators were excluded

| Upstream Regulator | Molecule Type | Activation | p-value of |
|---|---|---|---|
| CDKN1A | Other | -3.688 | 3.68E-17 |
| E2F1 | transcription regulator | 5 | 1.91E-15 |
| MYC | transcription regulator | 7.745 | 1.84E-14 |
| XBP1 | transcription regulator | 7.864 | 5.63E-14 |
| let-7 | microRNA | -7.67 | 2.62E-13 |
| MYCN | transcription regulator | 5.696 | 1.88E-12 |
| RB1 | transcription regulator | -4.904 | 1.91E-12 |
| CDKN2A | transcription regulator | -5.151 | 3.04E-12 |
| NFE2L2 | transcription regulator | 6.705 | 3.33E-10 |
| E2f | Group | 3.208 | 8.82E-10 |
| TBX2 | transcription regulator | 5.857 | 2.86E-09 |
| PTGER2 | G-protein coupled receptor | 5.092 | 4.28E-09 |
| EP400 | Other | 4.029 | 7.37E-09 |
| CCND1 | transcription regulator | 4.56 | 8.15E-09 |
| NUPR1 | transcription regulator | -9.207 | 3.79E-08 |
| miR-1 (and other miRNAs w/seed GGAAUGU) | mature microRNA | -7.472 | 1.92E-07 |
| E2F2 | transcription regulator | 2.5 | 2.75E-07 |
| RICTOR | Other | -7.97 | 4.30E-07 |
| Rb | Group | -4.619 | 5.80E-07 |
| KDM5B | transcription regulator | -5.879 | 3.53E-06 |

Supplemental Table 6b: Top 20 most significant upstream regulators predicted to be significantly altered in group B vs. group A samples in the **Jorissen cohort**, with absolute activation z-scores ≥ 2. Chemical upstream regulators were excluded.

| Upstream Regulator | Molecule Type | Activation z-score | p-value of overlap |
|---|---|---|---|
| TGFB1 | growth factor | 7.62 | 4.62E-43 |
| IFNG | cytokine | 11.73 | 9.13E-42 |
| TP53 | transcription regulator | 4.115 | 1.14E-41 |
| TNF | cytokine | 11.051 | 7.45E-33 |
| IL2 | cytokine | 6.836 | 3.86E-26 |
| STAT3 | transcription regulator | 6.512 | 1.81E-23 |
| IFNA2 | cytokine | 8.837 | 7.99E-23 |
| OSM | cytokine | 7.171 | 1.63E-22 |
| CD40LG | cytokine | 7.058 | 9.83E-22 |
| IL4 | cytokine | 5.298 | 4.38E-21 |
| CD3 | complex | -4.992 | 5.04E-21 |
| IL1B | cytokine | 10.491 | 1.23E-20 |
| HRAS | enzyme | 2.703 | 1.50E-20 |
| IL6 | cytokine | 8.821 | 8.31E-20 |
| HGF | growth factor | 5.829 | 1.13E-19 |
| APP | other | 4.657 | 1.50E-19 |
| Interferon alpha | group | 8.861 | 1.51E-19 |
| NFKBIA | transcription regulator | 4.071 | 2.99E-19 |
| Vegf | group | 7.696 | 2.77E-18 |
| CSF2 | cytokine | 8 | 3.87E-18 |

Supplemental Table 6c: Upstream regulators predicted to be significantly altered in **our cohort and the Jorissen cohort** in group B vs. group A samples, with absolute activation z-scores ≥ 2. Chemical upstream regulators are not shown.

| Upstream Regulator | Molecule Type | Activation | p-value of | Activation | p-value of |
|---|---|---|---|---|---|
| BAX | Transporter | 3.223 | 0.0103 | 3.471 | 9.67E-06 |
| CD28 | transmembrane receptor | -6.198 | 0.0313 | -3.741 | 4.54E-13 |
| KDM5B | transcription regulator | -5.879 | 3.53E-06 | -4.241 | 3.05E-07 |
| TSC22D1 | transcription regulator | 2.236 | 0.00386 | 2.449 | 0.00112 |
| E2F2 | transcription regulator | 2.5 | 2.75E-07 | 2.63 | 0.00495 |
| SREBF1 | transcription regulator | 3.145 | 0.00983 | 2.371 | 0.00225 |
| ATF4 | transcription regulator | 4.22 | 0.000396 | 3.736 | 0.000108 |
| FOXM1 | transcription regulator | 4.498 | 1.65E-05 | 3.503 | 0.000101 |
| E2F1 | transcription regulator | 5 | 1.91E-15 | 3.969 | 3.23E-08 |
| NFE2L2 | transcription regulator | 6.705 | 3.33E-10 | 4.295 | 5.58E-07 |
| XBP1 | transcription regulator | 7.864 | 5.63E-14 | 7.336 | 1.58E-10 |
| Irgm1 | Other | -4.101 | 0.00292 | -4.208 | 5.15E-06 |
| BID | Other | 2.121 | 0.0136 | 3.302 | 0.000496 |
| APP | Other | 2.403 | 0.00363 | 4.657 | 1.50E-19 |
| PRNP | Other | 2.714 | 0.034 | 2.283 | 2.34E-05 |
| RAB1B | Other | 2.864 | 0.00401 | 2.469 | 0.00657 |
| SCAP | Other | 3.59 | 0.00525 | 2.652 | 0.0429 |
| GAST | Other | 4.8 | 0.0156 | 3.616 | 0.00011 |
| CD24 | Other | 5.385 | 0.0145 | 2.853 | 4.10E-10 |
| let-7 | microRNA | -7.67 | 2.62E-13 | -2.755 | 5.97E-07 |
| miR-124-3p (and | mature microRNA | -8.019 | 3.20E-05 | -7.563 | 3.17E-12 |
| miR-1 (and other | mature microRNA | -7.472 | 1.92E-07 | -6.004 | 3.74E-12 |
| miR-16-5p (and | mature microRNA | -7.363 | 8.80E-06 | -4.226 | 1.12E-08 |
| miR-155-5p | mature microRNA | -5.855 | 0.00501 | -5.971 | 4.12E-15 |
| miR-30c-5p (and | mature microRNA | -5.81 | 0.00016 | -4.682 | 4.68E-09 |
| let-7a-5p (and other | mature microRNA | -5.531 | 0.0158 | -2.946 | 0.00713 |
| miR-291a-3p (and | mature microRNA | -4.991 | 0.0457 | -2.811 | 0.000176 |
| miR-145-5p (and | mature microRNA | -3.312 | 0.0471 | -4.289 | 8.14E-05 |
| miR-34a-5p (and | mature microRNA | -2.707 | 0.0207 | -2.88 | 2.14E-06 |
| PIM1 | Kinase | 2.113 | 0.0221 | 2.175 | 0.0018 |
| ATM | Kinase | 2.685 | 0.000793 | 2.635 | 1.93E-06 |
| ANGPT2 | growth factor | 5.468 | 0.000382 | 5.213 | 2.70E-10 |
| HGF | growth factor | 7.825 | 0.00285 | 5.829 | 1.13E-19 |
| caspase | Group | 2.538 | 0.016 | 2.55 | 0.0373 |
| Jnk | Group | 3.323 | 0.0293 | 5.8 | 8.19E-12 |
| PTGER2 | G-protein coupled receptor | 5.092 | 4.28E-09 | 2.702 | 0.0102 |
| CAT | Enzyme | -2.745 | 0.025 | -3.046 | 7.31E-12 |
| PLA2G2A | Enzyme | 2.228 | 0.0108 | 2.559 | 0.00036 |
| PIN1 | Enzyme | 2.534 | 0.0128 | 2.107 | 0.00232 |
| CD38 | Enzyme | 4.713 | 0.0189 | 5.897 | 5.13E-07 |
| IL3 | Cytokine | 3.316 | 0.0132 | 4.617 | 4.88E-08 |
| IL5 | Cytokine | 5.279 | 0.0306 | 7.804 | 1.99E-14 |
| CSF2 | Cytokine | 8.068 | 0.000137 | 8 | 3.87E-18 |
| CD3 | Complex | -7.035 | 0.00874 | -4.992 | 5.04E-21 |

Supplemental Table 7a: Top 20 most significantly different IPA diseases and functions group B vs. group A samples in our cohort. A p-value of overlap of 0.05 and |z-score| cutoff of 2 was used.

| Categories | Diseases or Functions | p-Value | Activation |
|---|---|---|---|
| Cell Cycle, DNA Replication, Recombination, and Repair | checkpoint control | 7.43E-16 | 3.38 |
| DNA Replication, Recombination, and Repair | DNA replication | 5.96E-14 | 2.439 |
| DNA Replication, Recombination, and Repair | repair of DNA | 1.05E-11 | 3.485 |
| Cell Cycle | cell cycle progression | 5.97E-11 | 3.578 |
| Infectious Disease | infection of cells | 7.81E-09 | 9.811 |
| Gene Expression, Protein Synthesis | translation of RNA | 1.02E-08 | -3.348 |
| Cell Cycle | M phase | 1.28E-08 | 3.17 |
| Gene Expression, Protein Synthesis | translation of mRNA | 1.93E-08 | -3.348 |
| Cellular Compromise, DNA Replication, Recombination, and Repair | damage of chromosomes | 2.05E-08 | -3.861 |
| DNA Replication, Recombination, and Repair | metabolism of DNA | 6.24E-08 | 3.059 |
| Cellular Compromise, DNA Replication, Recombination, and Repair | breakage of chromosomes | 1.74E-07 | -3.422 |
| Cell Cycle | interphase | 2.32E-07 | 3.233 |
| Cell Cycle | cycling of centrosome | 5.55E-07 | 2.759 |
| Infectious Disease | infection by RNA virus | 5.75E-07 | 10.075 |
| Infectious Disease | infection by HIV-1 | 5.77E-07 | 9.176 |
| Infectious Disease | infection of tumour cell lines | 6.62E-07 | 7.739 |
| Infectious Disease, Reproductive System Disease | infection of cervical cancer cell lines | 6.76E-07 | 7.737 |
| Cell Cycle, DNA Replication, Recombination, and Repair | S phase checkpoint control | 6.85E-07 | 2.23 |
| Cell Cycle, DNA Replication, Recombination, and Repair | checkpoint control of tumour cell lines | 7.64E-07 | 2.059 |
| Cell Cycle | senescence of cells | 1.17E-06 | -3.239 |

Supplemental Table 7b: Top 20 most significantly different IPA diseases and functions group B vs. group A samples in the Jorissen cohort. A p-value of overlap of 0.05 and |z-score| cutoff of 2 was used.

| Categories | Diseases or Functions | p-Value | Predicted | Activation |
|---|---|---|---|---|
| Cellular Growth and Proliferation | proliferation of cells | 6.09E-46 | Increased | 6.123 |
| Cellular Movement | cell movement | 2.13E-35 | Increased | 7.194 |
| Cellular Movement | migration of cells | 1.42E-33 | Increased | 7.233 |
| Cellular Movement | invasion of cells | 3.58E-32 | Increased | 4.663 |
| Hematological System | quantity of leukocytes | 1.71E-31 | Increased | 5.323 |
| Cell Death and Survival | cell death of immune cells | 8.35E-30 | Increased | 3.67 |
| Hematological System Development and Function, Tissue Morphology | quantity of blood cells | 1.50E-28 | Increased | 4.982 |
| Cell Death and Survival | cell death of blood cells | 1.52E-28 | Increased | 3.468 |
| Cancer | advanced malignant tumour | 2.44E-28 | Increased | 2.61 |
| Cancer | growth of tumour | 6.45E-28 | Increased | 3.179 |
| Infectious Disease | Viral Infection | 9.93E-28 | Increased | 5.851 |
| Cancer | metastasis | 3.59E-27 | Increased | 2.61 |
| Cellular Function and | function of blood cells | 7.51E-26 | Increased | 2.927 |
| Cellular Development, Cellular Growth and Proliferation, Hematological System Development and Function | proliferation of immune cells | 1.34E-25 | Increased | 2.709 |
| Cellular Function and Maintenance | function of leukocytes | 2.27E-25 | Increased | 2.715 |
| Cellular Development, Cellular Growth and Proliferation | proliferation of tumour cell lines | 8.51E-25 | Increased | 3.402 |
| Cellular Movement | cell movement of tumour cell lines | 4.11E-24 | Increased | 4.435 |
| Cellular Movement | cell movement of blood cells | 5.40E-24 | Increased | 8.081 |
| Cellular Development, Cellular Growth and Proliferation | proliferation of blood cells | 6.64E-24 | Increased | 2.627 |
| Cellular Movement | migration of blood cells | 9.14E-24 | Increased | 8.181 |

Supplemental Table 7c: IPA diseases and functions categories shared between subgroups in our cohort (columns 3&4) and that of the Jorissen cohort (columns 5&6). A p-value of overlap of 0.05 and |z-score| cutoff of 2 was used.

| Categories | Diseases or functions annotation | p-value | Activation z score | p-value (Jorissen ) | Activation z score. (Jorissen) |
|---|---|---|---|---|---|
| Protein Synthesis | metabolism of protein | 0.000524 | 2.722 | 4.07E-09 | 2.824 |
| Molecular Transport, Protein Trafficking | transport of protein | 0.0022 | 3.648 | 1.25E-11 | 2.364 |
| Cell Death and Survival | cell viability of tumour cell lines | 0.000651 | 7.672 | 3.49E-14 | 4.475 |
| Cell Death and Survival | cell survival | 0.00393 | 8.121 | 6.58E-22 | 7.171 |
| Cellular Growth and Proliferation | proliferation of cells | 0.0019 | 8.856 | 6.09E-46 | 6.123 |
| Infectious Disease | infection by lentivirus | 3.47E-06 | 9.31 | 4.27E-08 | 7.254 |
| Infectious Disease | HIV infection | 4.09E-06 | 9.419 | 6.95E-08 | 7.356 |
| Infectious Disease | infection by Retroviridae | 4.78E-06 | 9.423 | 5.37E-08 | 7.442 |
| Infectious Disease | infection of cells | 7.81E-09 | 9.811 | 1.49E-11 | 7.583 |
| Infectious Disease | infection by RNA virus | 5.75E-07 | 10.075 | 2.01E-10 | 7.491 |
| Infectious Disease | Viral Infection | 9.10E-05 | 10.224 | 9.93E-28 | 5.851 |

Supplemental Table 8. PARADIGM-derived abstract processes significantly altered (FDR ≤ 0.05, absolute median difference between subgroups ≥ 0.25) between RPMM-subgroups in our cohort as well as the Jorissen cohort.

| Integrated pathway level (IPL) | FDR | Subgroup diff. | FDR (Jorissen) | Subgroup diff. (Jorissen) |
|---|---|---|---|---|
| negative_regulation_of_DNA_binding_(abstract) | 6.31E-03 | 2.7 | 7.76E-16 | 2.6 |
| anoikis_(abstract) | 3.66E-02 | 2.2 | 3.68E-16 | 3.3 |
| protein_catabolic_process_(abstract) | 8.22E-04 | 1.9 | 3.81E-15 | 1.2 |
| G2/M_transition_DNA_damage_checkpoint_(abstract) | 2.26E-02 | 1.9 | 7.77E-15 | 1.6 |
| response_to_radiation_(abstract) | 4.74E-02 | 1.7 | 8.62E-17 | 2.3 |
| DNA_damage_checkpoint_(abstract) | 1.27E-03 | 1.6 | 1.99E-04 | 0.9 |
| regulation_of_transcription_(abstract) | 1.21E-03 | 1.6 | 7.59E-04 | 0.6 |
| prostaglandin_biosynthetic_process_(abstract) | 2.36E-02 | 1.3 | 3.58E-07 | 0.7 |
| spindle_assembly_(abstract) | 1.99E-02 | 1.2 | 1.37E-02 | 0.6 |
| regulation_of_mitotic_centrosome_separation_(abstract) | 3.72E-03 | 1.2 | 3.22E-02 | 0.3 |
| protein_folding_(abstract) | 2.24E-03 | 1.2 | 7.27E-04 | 0.9 |
| G1/S_transition_checkpoint_(abstract) | 8.83E-03 | 1.0 | 1.59E-03 | 0.4 |
| Golgi_organization_(abstract) | 8.32E-03 | 1.0 | 4.35E-02 | 0.6 |
| activation_of_caspase_activity_by_cytochrome_c_(abstract) | 2.64E-02 | 0.8 | 3.34E-16 | 1.0 |
| cell_cycle_arrest_(abstract) | 3.09E-02 | -1.2 | 2.08E-06 | -0.5 |
| ribosome_biogenesis_(abstract) | 3.24E-02 | -2.9 | 8.79E-13 | -2.7 |

Subgroup diff.: median difference in PARADIGM activity score between each subgroup. The first two columns represent the FDR and difference in medians for our cohort

Supplemental Table 9: comparison of canonical pathways with particular biological interest in B group vs. A group CRCs compared to MSI vs. MSS status in the same cohort.

| Ingenuity Canonical Pathways | -log(p-value) (MSI vs. MSS) | Down-regulated (MSI vs. MSS) | Up-regulated (MSI vs. MSS) | -log(p-value) (group B vs. A) | Down-regulated (group B vs. A) | Up-regulated (group B vs. A) |
|---|---|---|---|---|---|---|
| Antigen Presentation Pathway | 6.28 | 1/34 (3%) | 22/34 (65%) | 9.28 | 1/34 (3%) | 28/34 (82%) |
| Colorectal Cancer Metastasis Signaling | 4.65 | 46/230 (20%) | 67/230 (29%) | 5.27 | 20/230 (9%) | 89/230 (39%) |
| Production of Nitric Oxide and Reactive Oxygen Species in Macrophages | 3.61 | 36/179 (20%) | 64/179 (36%) | 3.52 | 18/179 (10%) | 64/179 (36%) |
| VEGF Signaling | 2.73 | 19/89 (21%) | 25/89 (28%) | 3.61 | 10/89 (11%) | 36/89 (40%) |
| Role of Pattern Recognition Receptors in Recognition of Bacteria and Viruses | 2.53 | 17/118 (14%) | 42/118 (36%) | 2.51 | 8/118 (7%) | 46/118 (39%) |
| IL-17 Signaling | 2.47 | 12/72 (17%) | 29/72 (40%) | 5.94 | 4/72 (6%) | 40/72 (56%) |
| IL-8 Signaling | 2.19 | 32/183 (17%) | 55/183 (30%) | 4.14 | 15/183 (8%) | 71/183 (39%) |
| Protein Ubiquitination Pathway | 1.32 | 40/251 (16%) | 90/251 (36%) | 6.54 | 21/251 (8%) | 101/251 (40%) |
| Role of BRCA1 in DNA Damage Response | 1.13 | 12/61 (20%) | 16/61 (26%) | 1.85 | 12/61 (20%) | 17/61 (28%) |
| Cell Cycle: G2/M DNA Damage Checkpoint Regulation | 7.53E-01 | 13/49 (27%) | 13/49 (27%) | 1.79 | 7/49 (14%) | 17/49 (35%) |
| ATM Signaling | 4.80E-01 | 9/59 (15%) | 18/59 (31%) | 2.77 | 6/59 (10%) | 25/59 (42%) |

Supplemental Table 10: Comparison of HLA genes significantly differentially expressed in a) MSI vs. MSS cancers and b) group B vs. group A CRCs of the Jorissen cohort.

| Probeset ID | Gene symbol | FDR (MSI vs. MSS) | FC (MSI vs. MSS) | FDR (group B vs. group A) | FC (group B vs. group A) | Relative subtype-specific effect |
|---|---|---|---|---|---|---|
| 201137_s_at | HLA-DPB1 | 3.0E-06 | 2.4 | 4.0E-14 | 3.6 | increased |
| 203932_at | HLA-DMB | 3.3E-09 | 2.3 | 3.1E-10 | 2.4 | increased |
| 205671_s_at | HLA-DOB | 3.7E-05 | 1.6 | 1.4E-02 | 1.3 | decreased |
| 208894_at | HLA-DRA | 4.0E-06 | 3.1 | 9.9E-13 | 5.2 | increased |
| 209480_at | HLA-DQB1 | 9.9E-03 | 1.8 | 6.4E-03 | 1.8 | decreased |
| 209823_x_at | HLA-DQB1 | 2.3E-06 | 2.3 | 1.2E-12 | 3.3 | increased |
| 210982_s_at | HLA-DRA | 2.0E-06 | 3.0 | 5.8E-13 | 4.7 | increased |
| 211654_x_at | HLA-DQB1 | 2.1E-05 | 2.1 | 5.5E-07 | 2.3 | increased |
| 211656_x_at | HLA-DQB1 | 1.1E-05 | 1.9 | 1.8E-10 | 2.4 | increased |
| 211990_at | HLA-DPA1 | 1.7E-06 | 2.8 | 4.0E-10 | 3.6 | increased |
| 211991_s_at | HLA-DPA1 | 5.7E-07 | 3.1 | 2.9E-12 | 4.4 | increased |
| 212998_x_at | HLA-DQB1 | 1.2E-05 | 2.7 | 1.4E-08 | 3.4 | increased |
| 212999_x_at | HLA-DQB1 | 1.5E-03 | 1.7 | 4.2E-04 | 1.7 | increased |
| 213537_at | HLA-DPA1 | 4.0E-06 | 2.6 | 1.9E-07 | 2.8 | increased |
| 213831_at | HLA-DQA1 | 8.2E-03 | 2.1 | 1.3E-05 | 3.0 | increased |
| 217362_x_at | HLA-DRB6 | 5.2E-07 | 1.7 | 4.0E-10 | 1.9 | increased |
| 217478_s_at | HLA-DMA | 7.8E-08 | 3.0 | 4.4E-10 | 3.4 | increased |
| 226878_at | HLA-DOA | 1.5E-05 | 1.9 | 2.2E-06 | 1.9 | increased |
| 236203_at | HLA-DQA1 | 8.2E-04 | 1.6 | 3.3E-03 | 1.5 | decreased |

Relative subtype-specific effect indicates a relative increase or decrease in the result comparing the CRC subtype-specific effect to the MSI-specific effect.

Supplemental Table 11a. Summary of differential gene expression analyses conducted for ***tumour*** samples showing the number of transcript clusters differentially expressed for each comparison made.

| Model specified | Number of samples/category | FDR $\leq$ 0.05, FC $\geq$ 2 |
|---|---|---|
| EF+ vs. EF– | 7 vs. 10 | 128 |
| FB-H vs. FB-L/N | 7 vs. 12 | 3 |
| afaC-H vs. afaC-L/N | 5 vs. 14 | 0 |
| afaC+ vs. afaC– | 13 vs. 8 | 0 |
| ClB+ vs. ClB– | 6 vs.13 | 0 |
| ETBF-H vs. ETBF L/N | 3 vs. 16 | 0 |
| ETBF+ vs. ETBF– | 10 vs. 9 | 0 |
| EPEC+ vs. EPEC– | 3 vs. 16 | 0 |

H: high-level infection; L: low-level infection; N: no infection; FB: Fusobacterium; EF: *E. faecali*s

Supplemental Table 11b. Genes differentially expressed in *E. faecalis*+ CRCs. The top 50 (of 128) transcriptclusters (sorted by FDR) are shown.

| Gene ID | Gene symbol | Gene Name | P value | FDR | FC |
|---|---|---|---|---|---|
| 8166906 | GPR34 | G protein-coupled receptor 34 | 2.6E-07 | 3.9E-03 | 2.4 |
| 8126784 | PLA2G7 | phospholipase A2, group VII (platelet-activating factor acetylhydrolase, plasma) | 4.3E-07 | 3.9E-03 | 2.7 |
| 8138289 | ETV1 | ets variant 1 | 5.3E-07 | 3.9E-03 | 2.3 |
| 7965410 | DCN | decorin | 2.5E-06 | 8.6E-03 | 3.9 |
| 8100541 | IGFBP7 | insulin-like growth factor binding protein 7 | 2.6E-06 | 8.6E-03 | 2.9 |
| 8101126 | CXCL10 | chemokine (C-X-C motif) ligand 10 | 2.6E-06 | 8.6E-03 | 5.8 |
| 8102792 | PCDH18 | protocadherin 18 | 3.1E-06 | 8.6E-03 | 3.7 |
| 7957737 | TMPO | thymopoietin | 3.1E-06 | 8.6E-03 | 2.1 |
| 7965403 | LUM | lumican | 4.1E-06 | 9.4E-03 | 6.1 |
| 8076292 | DNAJB7 | DnaJ (Hsp40) homolog, subfamily B, member 7 | 4.9E-06 | 9.4E-03 | -1.8 |
| 8125556 | HLA-DPA1 | major histocompatibility complex, class II, DP alpha 1 | 5.0E-06 | 9.4E-03 | 3.6 |
| 8017210 | AP1S2 | adaptor-related protein complex 1, sigma 2 subunit | 5.2E-06 | 9.4E-03 | 2.2 |
| 7898988 | CLIC4 | chloride intracellular channel 4 | 6.3E-06 | 1.0E-02 | 2.1 |
| 7919815 | CTSK | cathepsin K | 6.7E-06 | 1.0E-02 | 3.5 |
| 8178891 | HLA-DPA1 | major histocompatibility complex, class II, DP alpha 1 | 7.4E-06 | 1.1E-02 | 3.5 |
| 8001800 | CDH11 | cadherin 11, type 2, OB-cadherin (osteoblast) | 7.9E-06 | 1.1E-02 | 3.1 |
| 8094625 | KLHL5 | kelch-like family member 5 | 9.3E-06 | 1.1E-02 | 2.6 |
| 8160238 | PSIP1 | PC4 and SFRS1 interacting protein 1 | 9.4E-06 | 1.1E-02 | 1.7 |
| 8157890 | PBX3 | pre-B-cell leukemia homeobox 3 | 1.0E-05 | 1.2E-02 | 1.9 |
| 7926609 | BMI1 | BMI1 polycomb ring finger oncogene | 1.1E-05 | 1.2E-02 | 2.0 |
| 8145470 | DPYSL2 | dihydropyrimidinase-like 2 | 1.2E-05 | 1.2E-02 | 2.1 |
| 8105229 | PELO | pelota homolog (Drosophila) | 1.4E-05 | 1.4E-02 | 2.8 |
| 8180100 | HLA-DPA1 | major histocompatibility complex, class II, DP alpha 1 | 1.5E-05 | 1.4E-02 | 2.8 |
| 8140840 | STEAP4 | STEAP family member 4 | 1.6E-05 | 1.4E-02 | 2.4 |
| 8127563 | COL12A1 | collagen, type XII, alpha 1 | 1.9E-05 | 1.6E-02 | 4.1 |
| 8128007 | GJB7 | gap junction protein, beta 7, 25kDa | 2.0E-05 | 1.7E-02 | -1.6 |
| 8091032 | FOXL2 | forkhead box L2 | 2.3E-05 | 1.8E-02 | -1.7 |
| 8046895 | FAM171B | family with sequence similarity 171, member B | 2.5E-05 | 1.9E-02 | 2.9 |
| 8174322 | MORC4 | MORC family CW-type zinc finger 4 | 2.7E-05 | 2.0E-02 | 2.3 |
| 7981377 | ANKRD9 | ankyrin repeat domain 9 | 2.8E-05 | 2.0E-02 | -1.5 |
| 7957260 | GLIPR1 | GLI pathogenesis-related 1 | 2.8E-05 | 2.0E-02 | 2.8 |
| 8036324 | ZNF260 | zinc finger protein 260 | 3.1E-05 | 2.0E-02 | 2.3 |
| 8053882 | DUSP2 | dual specificity phosphatase 2 | 3.1E-05 | 2.0E-02 | -1.6 |

| | | | | | |
|---|---|---|---|---|---|
| 8121319 | SOBP | sine oculis binding protein homolog (Drosophila) | 3.1E-05 | 2.0E-02 | 2.1 |
| 8173732 | TAF9B | TAF9B RNA polymerase II, TATA box binding protein (TBP)-associated factor, | 3.2E-05 | 2.0E-02 | 2.1 |
| 8176263 | TAF9B | TAF9B RNA polymerase II, TATA box binding protein (TBP)-associated factor, | 3.2E-05 | 2.0E-02 | 2.1 |
| 8143040 | SLC35B4 | solute carrier family 35, member B4 | 3.8E-05 | 2.1E-02 | 1.7 |
| 8143054 | AKR1B1 | aldo-keto reductase family 1, member B1 (aldose reductase) | 3.9E-05 | 2.1E-02 | 2.1 |
| 8157605 | STOM | stomatin | 3.9E-05 | 2.1E-02 | 1.5 |
| 7936322 | GPAM | glycerol-3-phosphate acyltransferase, mitochondrial | 3.9E-05 | 2.1E-02 | 1.6 |
| 7959761 | FAM101A | family with sequence similarity 101, member A | 4.0E-05 | 2.2E-02 | -1.6 |
| 8042439 | ANTXR1 | anthrax toxin receptor 1 | 4.4E-05 | 2.3E-02 | 3.3 |
| 7930833 | KCNK18 | potassium channel, subfamily K, member 18 | 4.6E-05 | 2.3E-02 | -1.6 |
| 8089714 | LSAMP | limbic system-associated membrane protein | 4.7E-05 | 2.3E-02 | 2.1 |
| 7958913 | OAS2 | 2'-5'-oligoadenylate synthetase 2, 69/71kDa | 5.0E-05 | 2.3E-02 | 2.3 |
| 7912852 | EIF1AX | eukaryotic translation initiation factor 1A, X-linked | 5.0E-05 | 2.3E-02 | 1.7 |
| 8115234 | ANXA6 | annexin A6 | 5.1E-05 | 2.3E-02 | 1.7 |
| 8169473 | PLS3 | plastin 3 | 5.1E-05 | 2.3E-02 | 3.1 |
| 8138805 | CPVL | carboxypeptidase, vitellogenic-like | 5.6E-05 | 2.4E-02 | 2.0 |
| 8135734 | CPED1 | cadherin-like and PC-esterase domain containing 1 | 5.6E-05 | 2.4E-02 | 2.4 |

Supplemental Table 11c: Ingenuity Canonical Pathways significantly associated with *E. faecalis* colonisation in CRCs ($p \leq 0.05$). Boldface entries were also significant in the comparison between B vs. A group B CRCs.

| Ingenuity Canonical Pathways | -log(p-value) | Downregulated | Upregulated |
|---|---|---|---|
| Antigen Presentation Pathway | 2.88 | 0/33 (0%) | 15/33 (45%) |
| Hepatic Fibrosis / Hepatic Stellate Cell Activation | 2.48 | 22/191 (12%) | 44/191 (23%) |
| Human Embryonic Stem Cell Pluripotency | 2.27 | 23/129 (18%) | 29/129 (22%) |
| Leukocyte Extravasation Signaling | 2.06 | 15/190 (8%) | 48/190 (25%) |
| Agranulocyte Adhesion and Diapedesis | 1.98 | 17/169 (10%) | 38/169 (22%) |
| Allograft Rejection Signaling | 1.91 | 0/36 (0%) | 11/36 (31%) |
| T Helper Cell Differentiation | 1.71 | 4/62 (6%) | 13/62 (21%) |
| **Aryl Hydrocarbon Receptor Signaling** | **1.68** | **7/135 (5%)** | **41/135 (30%)** |
| Chondroitin Sulfate Biosynthesis (Late Stages) | 1.65 | 6/43 (14%) | 5/43 (12%) |
| L-dopachrome Biosynthesis | 1.60 | 1/1 (100%) | 0/1 (0%) |
| Glucocorticoid Receptor Signaling | 1.58 | 18/252 (7%) | 67/252 (27%) |
| **Antiproliferative Role of TOB in T Cell Signaling** | **1.57** | **0/26 (0%)** | **14/26 (54%)** |
| Role of Pattern Recognition Receptors in Recognition of Bacteria and Viruses | 1.56 | 11/116 (9%) | 28/116 (24%) |
| OX40 Signaling Pathway | 1.55 | 0/46 (0%) | 17/46 (37%) |
| Growth Hormone Signaling | 1.53 | 6/69 (9%) | 15/69 (22%) |
| Colorectal Cancer Metastasis Signaling | 1.53 | 27/228 (12%) | 50/228 (22%) |
| Cdc42 Signaling | 1.48 | 11/121 (9%) | 32/121 (26%) |
| Chondroitin Sulfate Biosynthesis | 1.41 | 8/51 (16%) | 6/51 (12%) |
| Axonal Guidance Signaling | 1.39 | 57/424 (13%) | 81/424 (19%) |
| Calcium-induced T Lymphocyte Apoptosis | 1.36 | 3/53 (6%) | 18/53 (34%) |
| Dermatan Sulfate Biosynthesis | 1.36 | 8/53 (15%) | 7/53 (13%) |
| Role of JAK2 in Hormone-like Cytokine Signaling | 1.34 | 2/32 (6%) | 5/32 (16%) |
| Granulocyte Adhesion and Diapedesis | 1.32 | 12/159 (8%) | 35/159 (22%) |
| Hypusine Biosynthesis | 1.30 | 1/2 (50%) | 0/2 (0%) |
| Cardiolipin Biosynthesis II | 1.30 | 1/2 (50%) | 0/2 (0%) |
