# Supplementary material for: Bacterially-Associated Transcriptional Remodelling in a Distinct Genomic Subtype of Colorectal Cancer Provides a Plausible Molecular Basis for Disease Development
Source: PLoS One. 2016 Nov 15;11(11):e0166282. doi: 10.1371/journal.pone.0166282 (PMC5112903; doi:10.1371/journal.pone.0166282)
Supplement: S2 Appendix — (PDF) [file pone.0166282.s002.pdf]

Array-based methylation and CIMP analysis

For whole-genome array-based methylation analysis, samples were bisulfite converted using the EZ DNA Methylation-Gold Kit™, as described for MSP-based detection of *MLH1* methylation; again bisulfite conversion efficiency was assessed using *MYOD* as a positive control. Bisulfite-converted DNA was whole genome amplified and enzymatically fragmented prior to hybridization to Illumina HumanMethylation 450k BeadChip arrays, according to the manufacturer's instructions (1). Arrays were scanned on an Illumina HiSeq 2000 using Illumina's iScan technology.

Raw data was extracted and overall quality was assessed using Illumina's GenomeStudio Data Analysis software. Further quality checks were performed using the R package minfi's 'qcreport' function (2). All samples passed quality control assessment.

Data was normalized using beta mixture quantile normalisation (BMIQ) (3) implemented in the R package watermelon (4). Next, sites were filtered using the IMA (5) filter function IMA.methy450PP, where probes that had ≥10% of samples with detection P-value > 0.05 were excluded, as well as probes on the X and Y chromosomes, and probes containing known SNP sites. The results were as follows: 11847 sites with missing values were removed, 90401 sites contained SNPs and were removed, 10417 sites on the X and Y chromosomes were removed; 372912 sites were retained from the original 485577 sites.

CIMP status was defined using the Hinoue et al. CIMP-defining marker panel *(B3GAT2, FOXL2, KCNK13, RAB31,* and *SLIT1*) that identifies CIMP+ (CIMP-H or CIMP-L) tumours with 100% sensitivity and 95.5% specificity, with 2.4% misclassification using the condition of DNA methylation of three or more markers with a ß-value threshold of ≥ 0.1. However, because the majority of probes mapping to the CIMP-marker panel had ß-values of ≥ 0.1 in the present study a ß-value threshold of 0.1 could not be used as a meaningful threshold to define CIMP status. This could be due to differences in array chemistries between the HumanMethylation27 BeadChip used by Hinoue et al. (who used a ß-value threshold of 0.1) and the HumanMethylation450k BeadChip used in our study. CIMP status was therefore evaluated by RPMM-based subtyping of probes mapping to CpG islands of the Hinoue et al. CIMP+ marker panel of genes (Supplemental Fig. 7). These ten samples (along with 37T, which was classified as CIMP-stable according to the clustering obtained) were the only samples where at least three of the five genes in the panel had gene-level methylation ß-values of ≥ 0.3 (gene-level

methylation ß-values were obtained by calculating the median ß-value of probes mapping to a particular gene).

PARADIGM pathway analysis

PARADIGM is a pathway-activity inference approach that infers patient-specific alterations in genes, complexes and abstract processes based on one or more 'omics' datasets as evidence (6). Data was prepared for input to PARADIGM by median-centering the $\log_2$ gene expression data across samples for each gene (6). Methylation beta values in the 1500 bp region upstream from the transcription start-site were summarized from probe-level beta values, for each gene, using the IMA package (function indexregionfunc) (5). These values were then inverted, since PARADIGM relates high values to increased gene expression, and low values to decreased gene expression (and promoter methylation is generally associated with decreased gene expression); the inverted values were median-centered across samples and used as input to PARADIGM alongside gene expression data. The online version of PARADIGM (https://dna.five3genomics.com) was used with default settings, except for increasing the log-likelihood percent threshold from 0.05 to 0.01% as recommended for smaller cohorts. Hierarchical clustering (Euclidian distance, complete linkage) was performed on the subset of IPLs where at least 75% of samples had absolute activation scores of ≥ 0.25 (6), which left 5334/17348 IPLs; two clear subsets were evident from the hierarchical clustering results, which were used for differential abundance testing.

References

1. Illumina. Infinium HD Assay Methylation Protocol Guide. 2011. page 1–247.

2. Aryee MJ, Jaffe AE, Corrada-Bravo H, Ladd-Acosta C, Feinberg AP, Hansen KD, et al. Minfi: a flexible and comprehensive Bioconductor package for the analysis of Infinium DNA methylation microarrays. Bioinformatics. 2014;30:1363–9.

3. Teschendorff AE, Marabita F, Lechner M, Bartlett T, Tegner J, Gomez-Cabrero D, et al. A beta-mixture quantile normalization method for correcting probe design bias in Illumina Infinium 450 k DNA methylation data. Bioinformatics. 2013;29:189–96.

4. Pidsley R, Y Wong CC, Volta M, Lunnon K, Mill J, Schalkwyk LC. A data-driven approach to preprocessing Illumina 450K methylation array data. BMC Genomics. 2013;14:293.

5. Wang D, Yan L, Hu Q, Sucheston LE, Higgins MJ, Am- CB, et al. IMA : An R package for high-throughput analysis of Illumina ' s 450K Infinium methylation data. Oxford Univ Press. 2012;1–3.

6. Vaske CJ, Benz SC, Sanborn JZ, Earl D, Szeto C, Zhu J, et al. Inference of patient-specific pathway activities from multi-dimensional cancer genomics data using PARADIGM. Bioinformatics. 2010;26:i237–45.
