# Supplementary figures and images for: Bacterially-Associated Transcriptional Remodelling in a Distinct Genomic Subtype of Colorectal Cancer Provides a Plausible Molecular Basis for Disease Development
Source: PLoS One. 2016 Nov 15;11(11):e0166282. doi: 10.1371/journal.pone.0166282 (PMC5112903; doi:10.1371/journal.pone.0166282)

**Supplemental  
Figure 1**

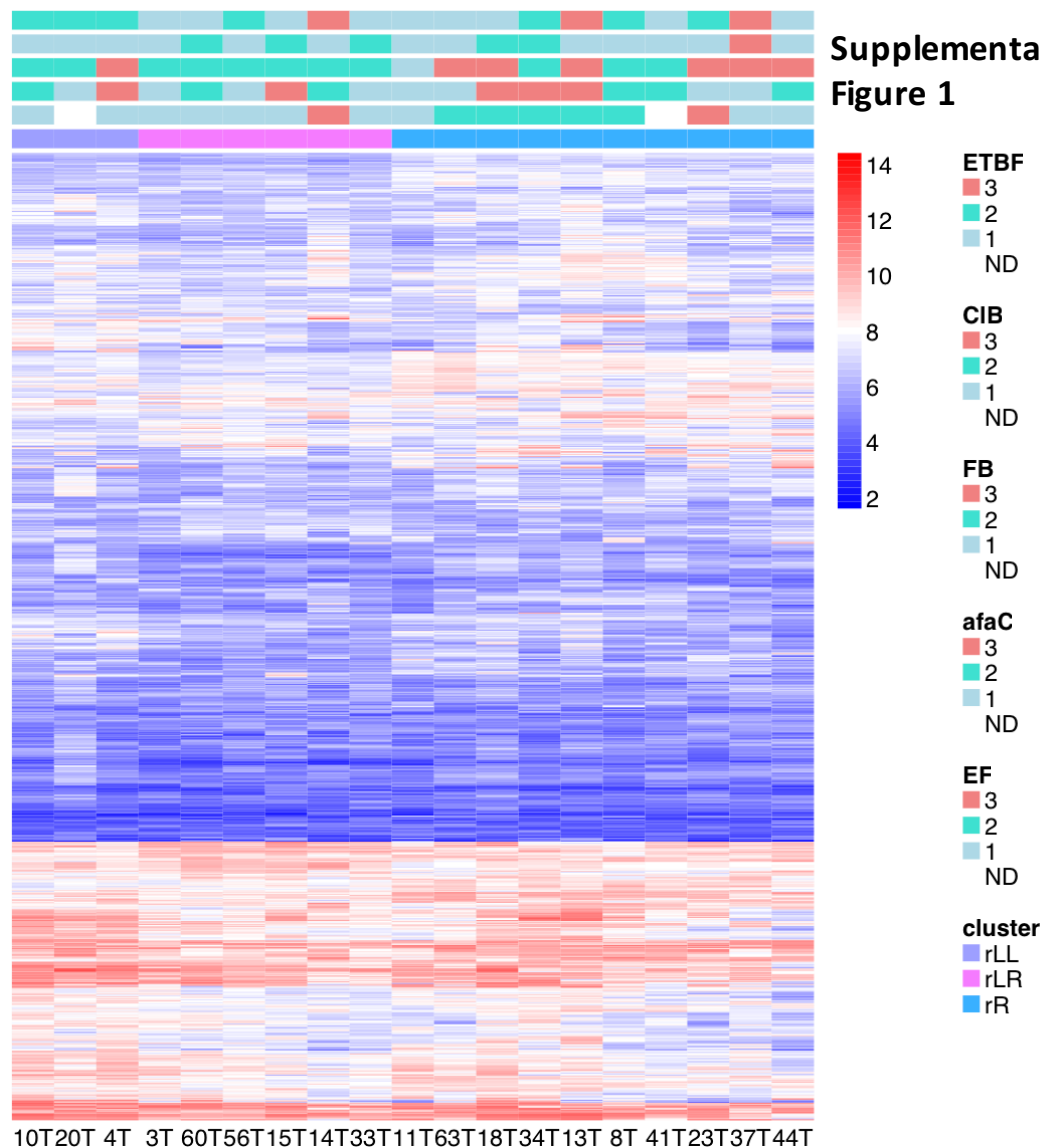

Supplement: S1 Fig — Levels of bacterial colonisation (described in the Methods section) are indicated on the figure legend, where 3: high- level colonisation, 2: low-level colonisation, 1: no colonisation. ETBF: Enterotoxigenic Bacteroides fragilis; ClB: ClB/pks+ E. coli, FB: Fusobacterium spp., afaC: afaC+ E. coli; EF: Enterococcus faecalis. The legend categories on the right are presented in the same order as the row annotations at the top of the graph. The scale on the right represents log2 expression values. (PDF) [file pone.0166282.s003.pdf]

# Supplemental Figure 2

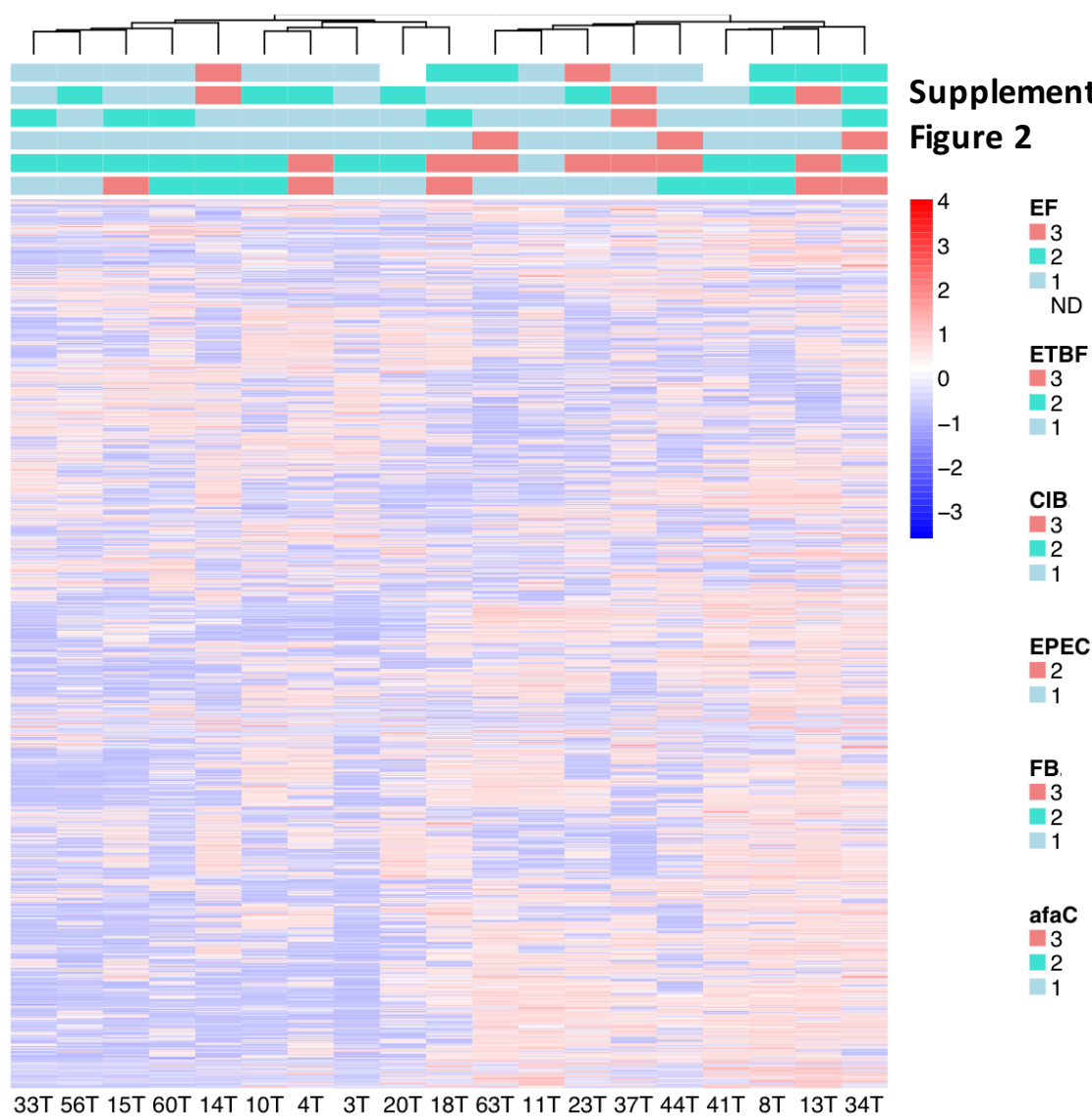

Supplement: S2 Fig — Two main clusters can be distinguished that are identical to the RPMM gene-expression clusters except for 18T. The scale on the right represents row-scaled expression values. EF.cat: E. faeclis colonisation category (1 = negative; 2 = low-level; 3 = high- level); ETBF.cat: ETBF colonisation category (1 = negative; 2 = low-level; 3 = high-level); ClB.cat: pks+ E. coli (1 = negative; 2 = low-level; 3 = high-level); EPEC.cat (1 = negative; 2 = positive); FB.cat: Fusobacterium colonisation category (1 = negative; 2 = low-level; 3 = high-level); afaC.cat: afaC+ E. coli (1 = negative; 2 = low-level; 3 = high-level); ND: not determined. (PDF) [file pone.0166282.s004.pdf]

# Supplemental Figure 4

de Sousa subgroups

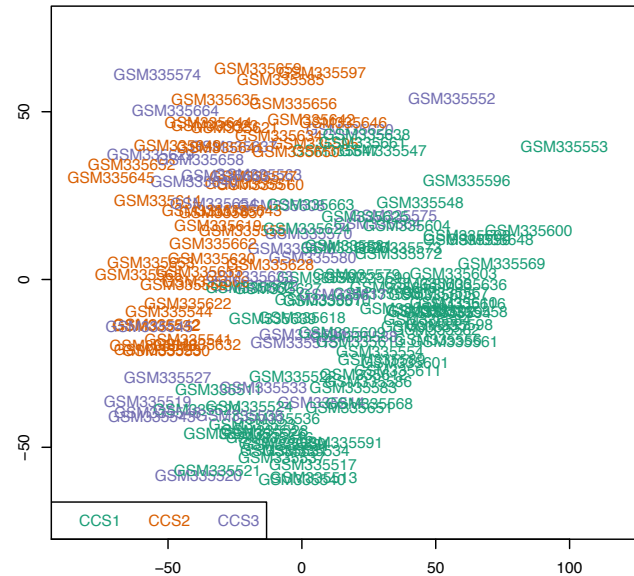

Sadanandam subgroups

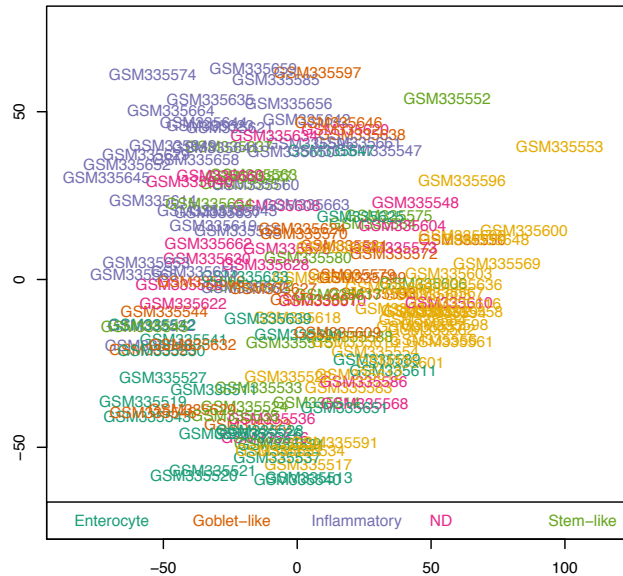

RPMM subgroups

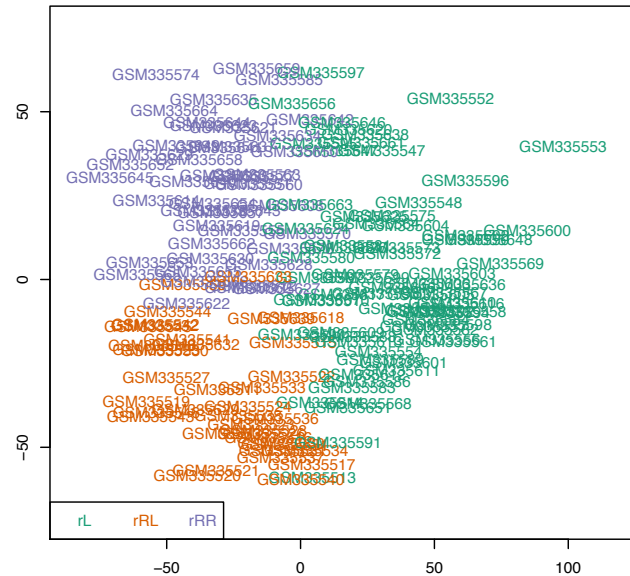

Supplement: S4 Fig — The three figures are identical apart from the annotation colours used, where samples have been coloured by the De Sousa E Melo (left), Sadanandam (middle) or RPMM (right) subgroups. (PDF) [file pone.0166282.s006.pdf]

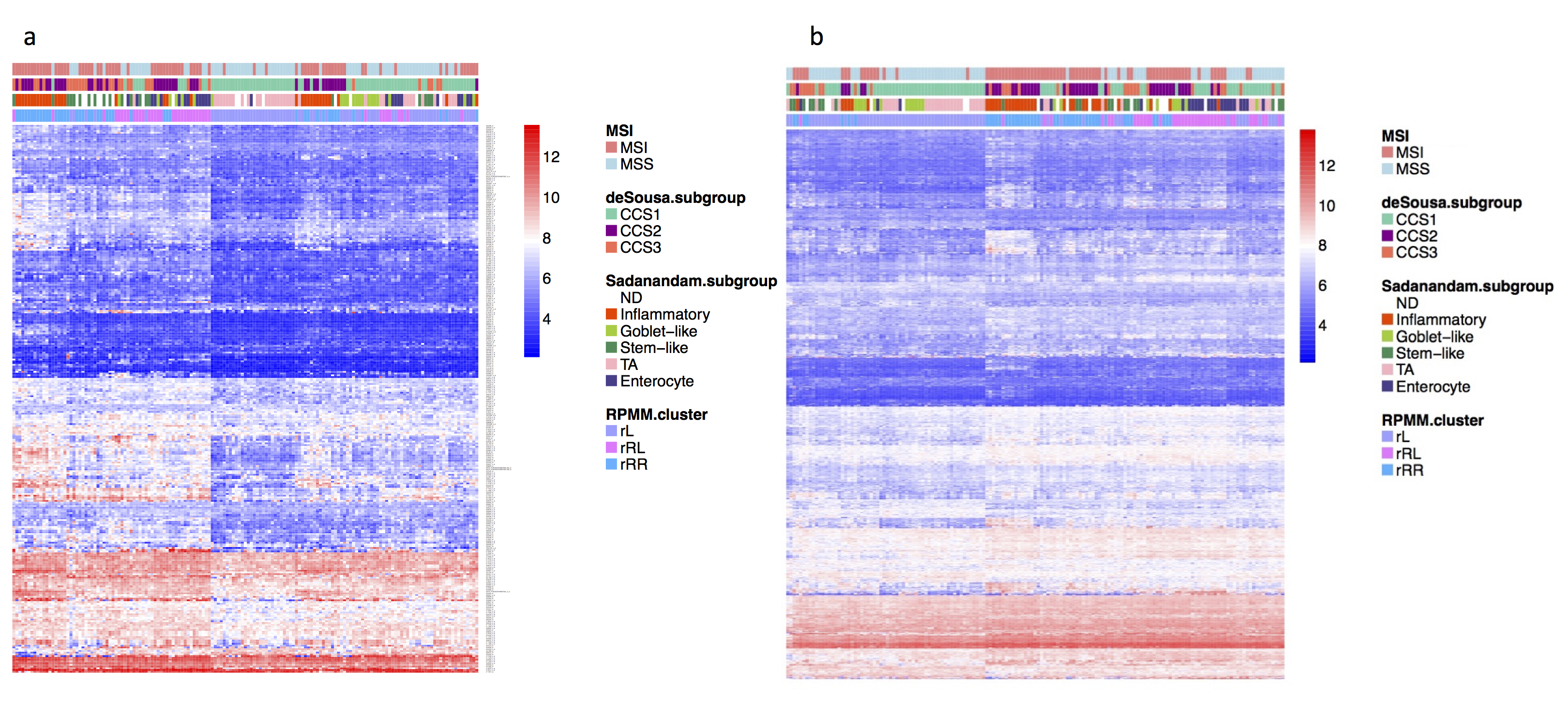

Supplement: S6 Fig — a) Hierarchical clustering of the 218 genes differentially expressed between groups A and B that were classified under the IPA diseases and functions category Bacterial Infection; b) Hierarchical clustering of the 912 genes differentially expressed between groups A and B that were classified under the IPA diseases and functions category Viral Infection. (TIFF) [file pone.0166282.s008.tiff]
