# Supplementary material for: Bacterially-Associated Transcriptional Remodelling in a Distinct Genomic Subtype of Colorectal Cancer Provides a Plausible Molecular Basis for Disease Development
Source: PLoS One. 2016 Nov 15;11(11):e0166282. doi: 10.1371/journal.pone.0166282 (PMC5112903; doi:10.1371/journal.pone.0166282)

Supplemental Figure 3

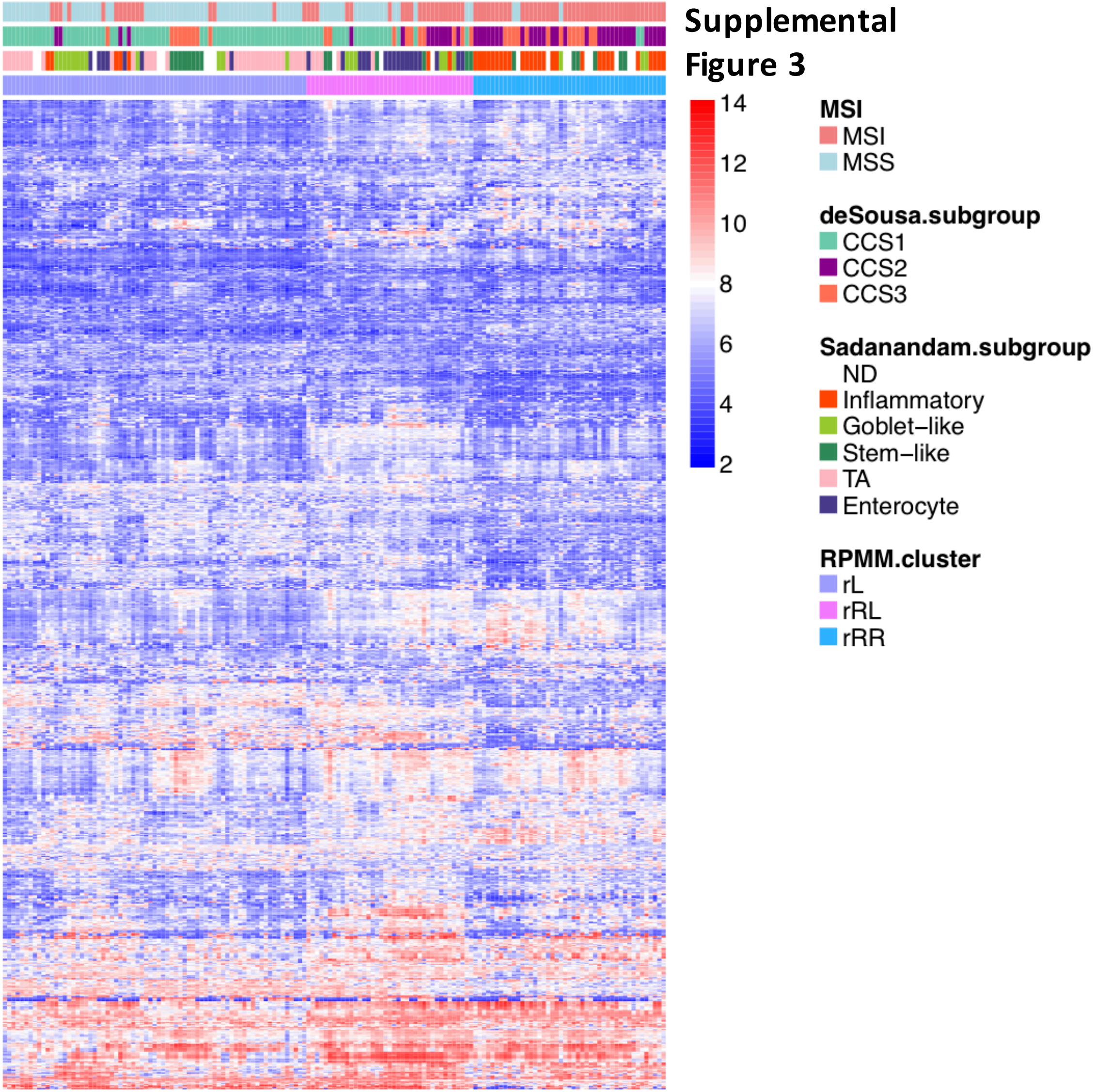

Supplement: S3 Fig — RPMM clusters are displayed alongside the previously established de Sousa and Sadanandam subtypes and MSI-status for each sample. Here, only the top 1000 most variable probes are displayed, although clustering was conducted on the top quartile most variable probes. The legend categories on the right are presented in the same order as the row annotations at the top of the graph. The scale on the right represents log2 expression values. The rRL and rRR clusters together are referred to as group B, while the rL cluster is referred to as group A. (PDF) [file pone.0166282.s005.pdf]
