# Supplementary material for: Bacterially-Associated Transcriptional Remodelling in a Distinct Genomic Subtype of Colorectal Cancer Provides a Plausible Molecular Basis for Disease Development
Source: PLoS One. 2016 Nov 15;11(11):e0166282. doi: 10.1371/journal.pone.0166282 (PMC5112903; doi:10.1371/journal.pone.0166282)

Supplemental Figure 5

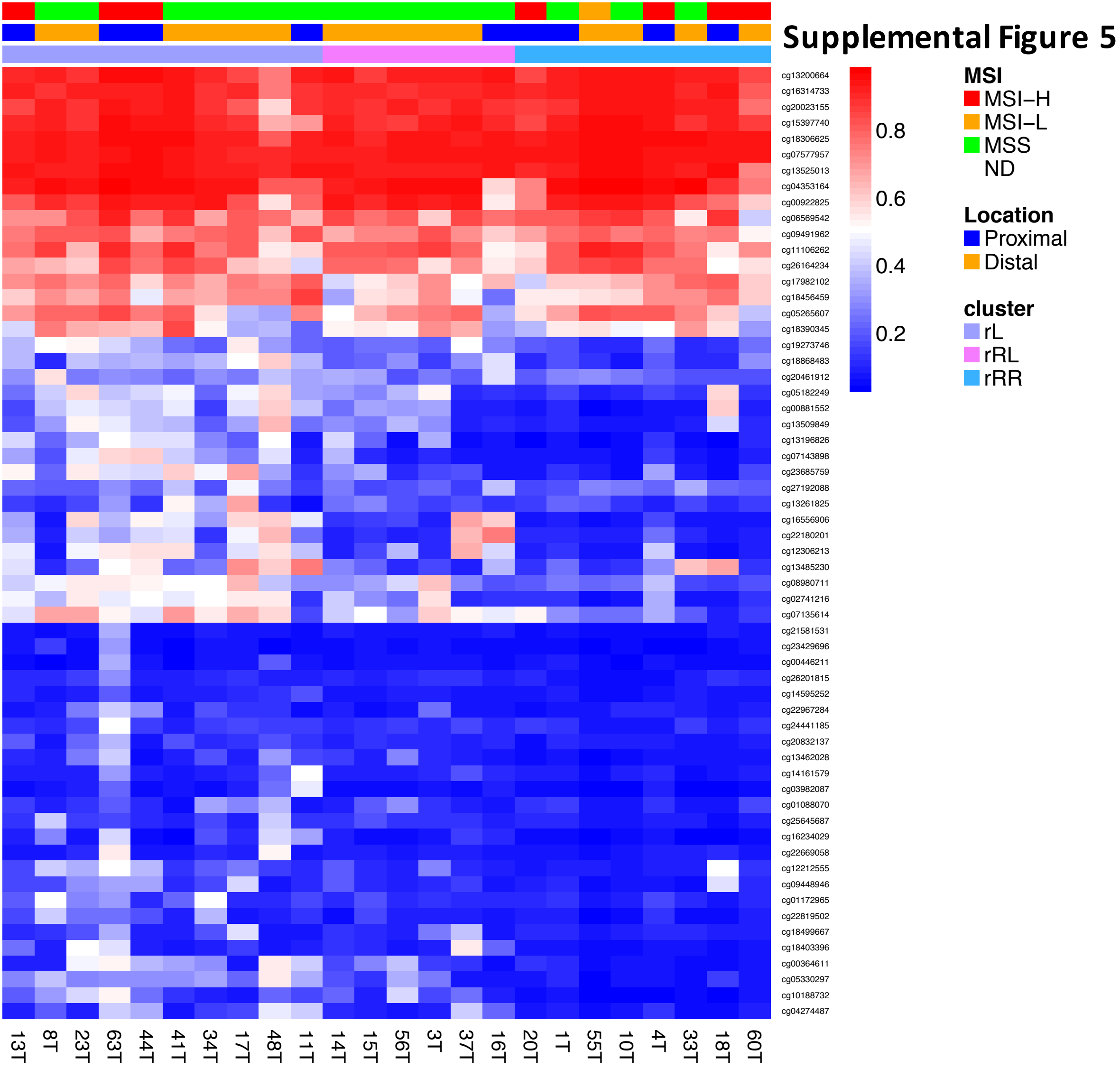

Supplement: S5 Fig — RPMM-based clustering of probes mapping to CpG islands in the Hinoue CIMP marker panel (B3GAT2, FOXL2, KCNK13, RAB31, and SLIT1). Samples in the rL cluster are considered to be CIMP+. The legend categories on the right are in the same order as the row annotations at the top of the graph. The scale on the right of the heatmap indicates beta values (0–1). Patients 13, 18, 20 and 4 were diagnosed with HNPCC. (PDF) [file pone.0166282.s007.pdf]
